# Coordination-induced bond weakening of water at the surface of an oxygen-deficient polyoxovanadate cluster†

**DOI:** 10.1039/d2sc04843d

**Published:** 2022-10-11

**Authors:** Shannon E. Cooney, Alex A. Fertig, Madeleine R. Buisch, William W. Brennessel, Ellen M. Matson

**Affiliations:** Department of Chemistry, University of Rochester Rochester NY 14627 USA afertig@u.rochester.edu matson@chem.rochester.edu

## Abstract

Hydrogen-atom (H-atom) transfer at the surface of heterogeneous metal oxides has received significant attention owing to its relevance in energy conversion and storage processes. Here, we present the synthesis and characterization of an organofunctionalized polyoxovanadate cluster, (calix)V_6_O_5_(OH_2_)(OMe)_8_ (calix = 4-*tert*-butylcalix[4]arene). Through a series of equilibrium studies, we establish the BDFE(O–H)_avg_ of the aquo ligand as 62.4 ± 0.2 kcal mol^−1^, indicating substantial bond weaking of water upon coordination to the cluster surface. Subsequent kinetic isotope effect studies and Eyring analysis indicate the mechanism by which the hydrogenation of organic substrates occurs proceeds through a concerted proton–electron transfer from the aquo ligand. Atomistic resolution of surface reactivity presents a novel route of hydrogenation reactivity from metal oxide surfaces through H-atom transfer from surface-bound water molecules.

## Abstract 2

Coordination-induced bond weakening facilitates H-atom transfer from polyoxovanadate surface.

## Graphical abstract

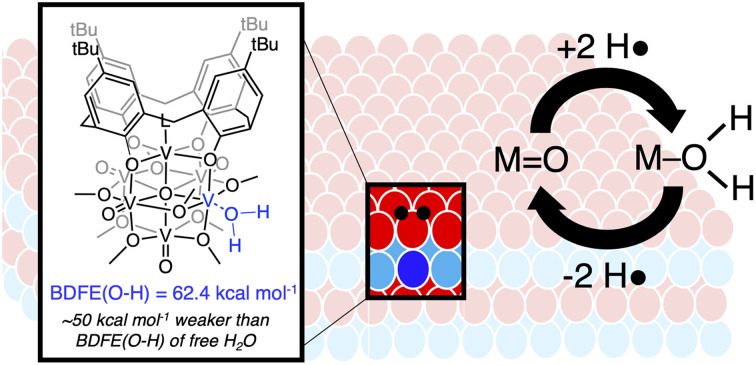

## Introduction

Manipulation of the reactivity of protons and electrons at electrochemical interfaces is integral for the development of improved energy storage and conversion processes. This fact renders an understanding of the basic science underlying proton-coupled electron transfer (PCET) at redox active surfaces important for the development of efficient and sustainable strategies for the production of commodity chemicals. One class of compounds that has risen to prominence in the area of PCET reactions are heterogeneous metal oxides (MO_*x*_). These materials are ubiquitous in the activation of small molecules (*e.g.* O_2_, CO_2_, N_2_), and also have been reported to facilitate the direct utilization of H_2_ as a chemical fuel (*e.g.* water electrolyzers, fuel cells).^1–5^

**Fig. 1:**
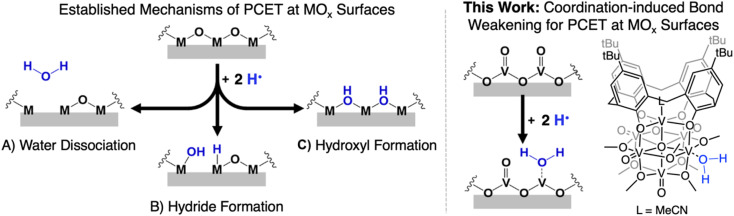
(Left) previously established mechanisms for the hydrogenation of the surface of metal oxide materials: (A) formation of an aquo ligand that subsequently dissociates from the MO_*x*_ surface resulting in the formation of an O-atom defect site; (B) formation of metal hydrides; (C) formation of bridging hydroxyl ligands. (right) Proposed mechanism in which hydrogenation results in the formation of a reactive aquo ligand that remains coordinated to the MO_*x*_ surface and the POV-alkoxide cluster used to model this mode of reactivity.

Despite the prevalence of MO_*x*_ in energy storage and conversion schematics involving the manipulation of hydrogen atom (H-atom) equivalents (e^−^/H^+^), there remain significant challenges in obtaining an atomistic understanding of reaction pathways by which H-atoms are transferred from the surface of MO_*x*_ to small molecule substrates. Mechanistic analysis is rendered particularly challenging due to difficultly in distinguishing between reactivity mediated by surface and inserted H-atom equivalents of extended MO_*x*_.^6–8^ Furthermore, the lack of uniformity by which H-atom equivalents interact with the surface of MO_*x*_ results in a wide variety of interpretations as to the mechanism of transfer of protons and electrons. Indeed, the inhomogeneity of MO_*x*_ surfaces provokes questions related to the role of bridging *vs.* terminal oxido moieties in H-atom uptake and transfer reactions, as well as multi-site PCET invoking surface ligand centred proton-transfer processes (Fig. 1).^9,10^

To experimentally model mechanisms of PCET at MO_*x*_ surfaces, researchers have turned to the investigation of their molecular analogues.^11–16^ One class of compounds that is particularly well-suited for these studies is polyoxometalates (POMs). POMs are three-dimensional MO_*x*_ assemblies composed of early transition metal oxyanions linked together by edge- or face-sharing oxide ligands. Broadly speaking, POMs possess similar surface morphologies to their extended state congeners, however, these clusters limit their reactivity with H-atom equivalents to surface bound oxide ligands (*i.e.* no intercalation). While proton-responsive redox properties have been reported for POMs,^17^ there remain limited examples in which thermodynamics or kinetics of PCET has been explored. The few studies that have investigated PCET at POM surfaces have revealed that the uptake of H-atom equivalents occurs primarily at bridging oxide positions.^12,18^

An alternative mechanism for the generation of reactive H-atom equivalents at MO_*x*_ surfaces invokes coordination-induced bond weakening of O–H moieties upon association of water to a surface oxygen-atom (O-atom) vacancy (Fig. 1). In this scenario, a coordinatively unsaturated, reduced metal centre at the surface of the material binds to water, resulting in a weakening of the O–H bond of the substrate. This phenomenon has been well-documented in the reactivity of water with reduced, mononuclear transition metal complexes,^19–27^ however, to date, has not been invoked in extended solids. This is despite the fact that surfaces of reducible metal oxide materials are riddled with these defects sites.^28–31^ As a result, our understanding as to how coordination-induced bond weakening impacts the design criteria for the formation of reactive H-atom equivalents at the surface of MO_*x*_ systems from “green” substrates (*e.g.* H_2_O, NH_3_) remains limited.

While touted as excellent models for studies investigating the surface reactivity of extended MO_*x*_ materials, the isolation of POMs possessing single O-atom vacancy is exceedingly rare. Early examples of the formation of O-atom defect sites in “heteropoly brown” tungsten oxide assemblies emerged from the work of Launay and Pope and Piepgrass. In these studies, reduction of the polyoxotungstate in the presence of acid results in the formation of the 6e^−^/6H^+^ reduced assembly [W^VI^_9_(W^IV^OH_2_)_3_].^32–35^ Despite the observation of aquo ligands bound to the reduced tungsten(iv) centres, H-atom transfer reactivity is not observed. Instead, the aquo ligands are displaced in organic solvents by oxygenated substrates, resulting in reoxidation of the cluster *via* reductive cleavage of E=O bonds (*e.g.*, O=AsR_3_, O=NPh).^36^ Our group has also reported O-atom vacancy formation in a series of reduced polyoxovanadate-alkoxide clusters (POV-alkoxide);^13,37–43^ most relevant to the studies reported here, we have described defect formation at the surface of the Lindqvist ion following the transfer of two H-atom equivalents to a terminal vanadyl site.^13^ However, it was found that the affinity of the reduced V^III^ ion toward acetonitrile results in rapid displacement of the transiently generated surface aquo ligand, preventing analysis of the surface bound water adduct.

Herein, we report the isolation of the first aquo complex of a POV-alkoxide cluster. This unique compound is accessed *via* addition of two H-atom equivalents to a terminal oxido ligand of a surface vanadyl in a calix-functionalized hexavanadate cluster, (calix)V_6_O_6_(MeCN)(OMe)_8_, (calix)V_6_O_6_. Quantification of the strength of the resultant O–H bonds of the aquo ligand is determined through a series of equilibrium studies. Results reveal that coordination of the aquo ligand to the surface of the cluster at an oxygen atom point defect results in significant weakening of the O–H bonds of water (∼50 kcal mol^−1^), rendering them reactive toward H-atom transfer. Further insight into the mechanism of delivery of H-atom equivalents to organic substrates is obtained through kinetic isotope effect studies and Eyring analysis. Our results indicate that the hydrogenation of the organic substrate relies on a rate determining concerted proton–electron transfer (CPET) step, as opposed to a stepwise transfer of protons and electrons. Our results indicate that the coordination of water to reactive surface defect sites may play a critical role as a source of environmentally benign H-atoms in hydrogenation schematics.

## Results and discussion

Previously, our research group has reported changes in the electrochemical properties of the polyoxovanadate-alkoxide cluster following surface-functionalization of the assembly with a calix ligand.^44^ The cyclic voltammogram of (calix)V_6_O_6_ possesses three quasi-reversible redox events, shifted by approximately +0.30 V in comparison to its non-substituted congener, [V_6_O_6_(OMe)_12_(MeCN)]^0^ (MeCN = acetonitrile). Curious as to whether the changes in the redox potentials would influence the reactivity of terminal vanadyl ligands with H-atoms, we set out to investigate the reactivity of (calix)V_6_O_6_ with dihydrophenazine (DHP). DHP has been established to have weak N–H bonds (*e.g.* BDFE(N–H)_avg_ of DHP in MeCN = 58.9 kcal mol^−1^), rendering this substrate a convenient entry point for our work.

To date, HAT reactions with POV-alkoxide clusters have been conducted in MeCN. The historical selection of this solvent is due to the breadth of reported thermochemical data (*e.g.* BDFE(E–H) values, p*K*_a_ values, reduction potentials) for organic substrates.^45–47^ This allows for facile benchmarking of the reactivity of surface oxido moieties with E–H bonds. Unfortunately, (calix)V_6_O_6_ has limited solubility in MeCN, necessitating studies be performed in an alternative solvent. For this work, we elected to use tetrahydrofuran (THF) as solvent.

**Scheme 1:**
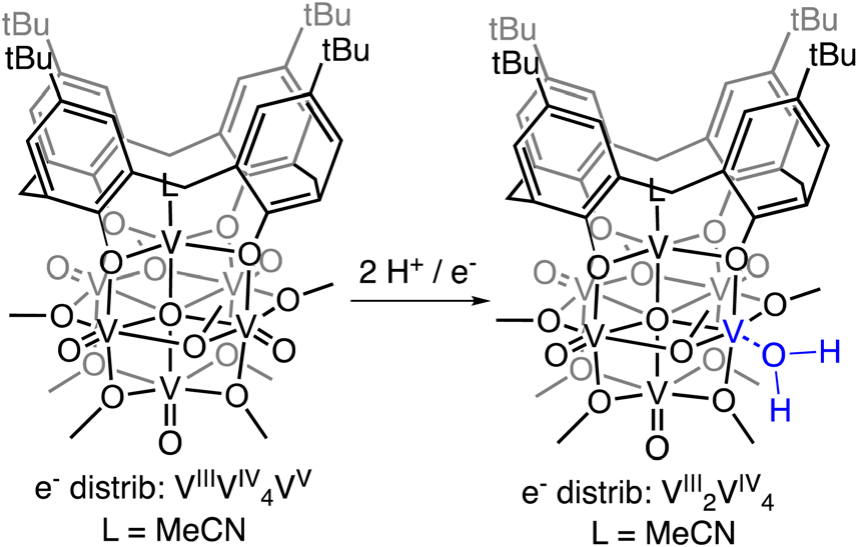
Synthesis of (calix)V_6_O_5_(OH_2_) by H-atom transfer.

Addition of 1 equiv. of DHP to (calix)V_6_O_6_ at room temperature in THF results in a colour change from brown to red over 1 h (Scheme 1). Analysis of the reaction mixture by ^1^H NMR spectroscopy reveals formation of a new product, with four paramagnetically shifted and broadened resonances (*δ* = 28.71, 26.45, −5.10, and −11.61 ppm). Additional signals corresponding to the calix ligand are located in the diamagnetic region of the spectrum (Fig. S1†). The ^1^H NMR spectrum of the product is substantially different from that of the starting material, (calix)V_6_O_6_ (Fig. S2†). In particular, two new, up-field resonances are observed. Previous reports from our laboratory have concluded that similar, negatively-shifted signals correspond to protons of methoxide ligands adjacent to a reduced vanadium centre embedded within the cluster core upon O-atom vacancy formation.^40,41,43,48^ Indeed, we have recently reported the reduction of an oxidized form of the calix-substituted POV-alkoxide cluster, [(calix)V_6_O_7_(OMe)_8_]^0^, by oxygen atom transfer to a tertiary phosphine; the product possesses a similar pattern of signals to that described here, albeit with resonances that possess different chemical shifts due to the differing oxidation state distribution of vanadium ions of the starting materials (e^−^ distrib. [(calix)V_6_O_7_(OMe)_8_ = V^IV^_4_V^V^_2_] *vs.* e^−^ distrib. (calix)V_6_O_6_ = V^III^V^IV^_4_V^V^).^49^

Further support for the reduction of the cluster core was obtained through analysis of the product by electronic absorption spectroscopy (Fig. S3†). This characterization technique has been well established by our group and others to report on the degree of reduction of the cluster core.^50–55^ Comparison of the electronic absorption spectra of the product of H-atom transfer to (calix)V_6_O_6_ reveals loss of an intervalence charge transfer band (V^IV^ → V^V^). This observation is consistent with formation of the desired oxygen-deficient product, as vacancy formation is associated with the reduction of the single V^V^ centre in (calix)V_6_O_6_ to V^III^. The product possesses weak absorptions at 417 nm (*ε* = 234 M^−1^ cm^−1^) and 521 nm (*ε* = 155 M^−1^ cm^−1^); similar transitions have been reported for the cis, di-vacant POV-alkoxide cluster, [V_6_O_5_(OMe)_12_(MeCN)_2_]^0^ (*λ* = 426 nm, *ε* = 379 M^−1^ cm^−1^; *λ* = 539 nm, *ε* = 184 M^−1^ cm^−1^). Similarities in the electronic structure between the product and [V_6_O_5_(OMe)_12_(MeCN)_2_]^0^ is consistent with the fact that H-atom transfer results in the formation of second defect site at the surface of the cluster.

**Fig. 2:**
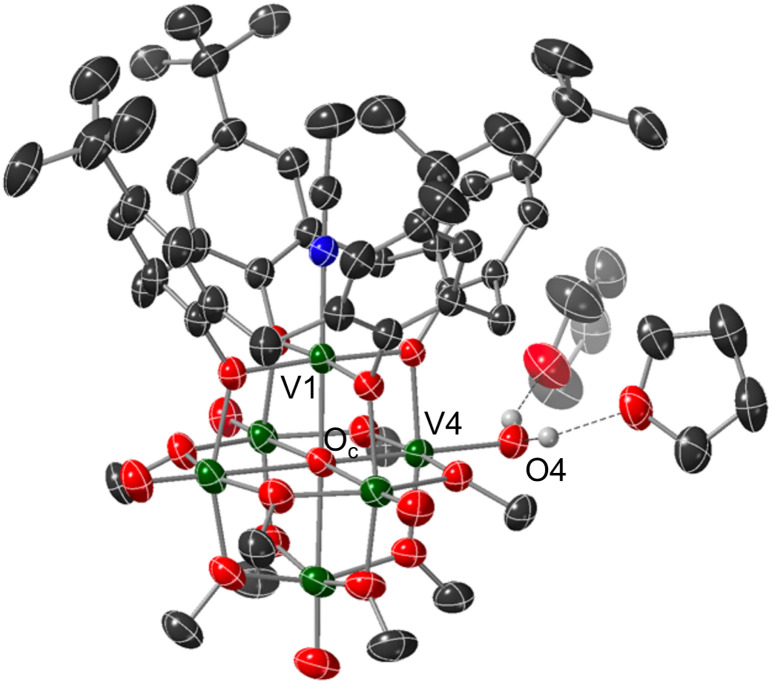
Molecular structure of (calix)V_6_O_5_(OH_2_) shown with 30% probability ellipsoids. Selected solvent molecules and H-atoms have been removed for clarity. Key: dark green ellipsoids: V; red ellipsoids: O; dark grey ellipsoids: C; blue ellipsoids: N; white spheres: H.

Crystals suitable for structural analysis by single crystal X-ray diffraction were grown *via* slow diffusion of pentane into a concentrated solution of the product in THF (Fig. 2 and Table 1). Refinement of the data revealed formation of the expected reduced assembly, [(calix)V_6_O_5_(OH_2_)(OMe)_8_] ((calix)V_6_O_5_(OH_2_)), with the new, “oxygen-deficient” vanadium centre positioned *cis* to the calix-bound vanadium ion. The asymmetric unit contains one-half of a vanadium cluster on a crystallographic mirror plane. Bond valence sum calculations obtained from the V–O bond lengths of structurally unique vanadium centres confirm the spectroscopically proposed oxidation state assignments of metals contained within the cluster core (V^III^_2_V^IV^_4_, Table S2†). The aquo ligand bound to the reduced vanadium centre is stabilized by hydrogen bonding interactions with two THF solvent molecules. Notably, the O4⋯O(THF) distances of 2.665(9) and 2.601(15) Å are quite short, suggesting the existence of strong H-bonding interactions between the coordinated aquo ligand and solvent.^56^

**Table 1:** Selected bond distances and angles in (calix)V_6_O_6_ and (calix)V_6_O_5_(OH_2_)

Bond	(calix)V_6_O_6_	(calix)V_6_O_5_(OH_2_)
V1–N1	2.105(2) Å	2.0676(17)
V1–O_c_	2.0676(17) Å	2.058(3) Å
V4–O4	—	2.052(2) Å
V4–O_c_	—	2.110(3) Å
V_e_=O_t_ (avg)	1.5938 Å	1.602 Å
V_*n*_–O_c_ (avg)	2.3586 Å	2.382 Å
O4⋯O(THF)	—	2.601(15), 2.665(9) Å
O4–H4⋯O(THF)	—	150(5)°, 154(5)°

Substantial elongation of the V4–O4 bond (2.052(4) Å) from that of the starting material (average V=O_t_ bond length *cis* to calix ligand = 1.594 Å) is observed, consistent with reduction of the vanadyl ion to a vanadium(iii) aquo. Indeed, the V4–O4 bond length resembles values reported previously for molecular vanadium(iii) aquo complexes (1.967(3)–2.086(2) Å).^57,58^ The independent refinement of the H-atoms of the aquo ligand (one unique) identified in the difference Fourier map further supports the assignment of an aquo ligand bound to the surface of the assembly.

While by no means is the formation of a vanadium(iii) aquo moiety rare,^57,59–64^ this result is significant within the context of our work, given its relevance as the direct product of multielectron/multiproton coupled transfer to a terminal vanadyl moiety. Our previous studies on CPET reactions for the generation of O-atom defects at the surface of POV-alkoxides have invoked transient formation of the reduced aquo complex.^13^ However, in the presence of MeCN, this aquo ligand readily dissociates from the surface, resulting in the isolation of the MeCN-bound assembly, [V_6_O_6_(MeCN)(OMe)_12_]^*n*^ (*n* = −1, 0, +1). Despite the basic character of the oxygen atom of THF, under the reaction conditions reported here, the solvent does not displace the aquo ligand from the surface of the assembly. Instead, THF functions as a hydrogen bond acceptor, likely stabilizing (calix)V_6_O_5_(OH_2_) through the formation of strong intermolecular hydrogen bonding interactions.

Previous work from our group has shown that the BDFE(O–H) of reduced POV-alkoxide clusters can be approximated by monitoring the extent to which H-atom transfer occurs between various organic reagents and a cluster.^12,13^ The observation of quantitative reduction of the POV suggests that the BDFE(O–H) of the cluster is sufficiently larger than that of DHP, establishing an effective minimum value of the BDFE(O–H)_avg_ for the reduced cluster in THF. However, despite the fact that there exists a substantial library of known thermochemical parameters of PCET reagents, the BDFE(N–H)_avg_ for DHP is not reported in THF. In order to circumvent this obstacle, we turned to alternative methods for obtaining the free energy of the N–H bonds of DHP in THF. While THF has traditionally been underutilized in PCET chemistry, the thermodynamics of PCET reagents in MeCN are well established, including the BDFE(N–H)_avg_ for DHP: 58.7 kcal mol^−1^.^47^ We are able to calculate the BDFE(N–H)_avg_ for DHP in THF using eqn (1), where 
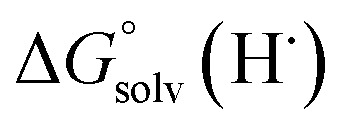
 (solv = MeCN, THF; 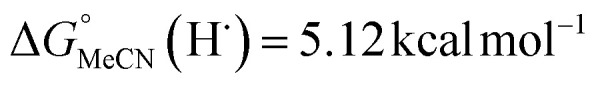; 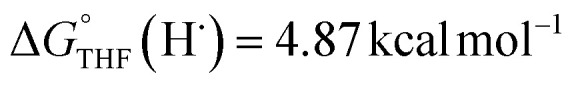)^65^ is the free energy of solvation for a hydrogen radical in the solvent of interest, and 
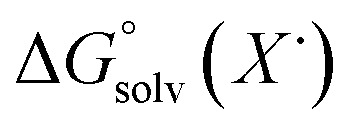
 and 
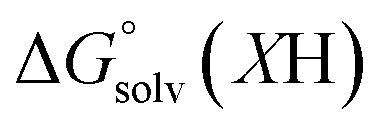
 (solv = MeCN, THF) are the free energy of solvation for the radical and the reduced organic substrate, respectively.1
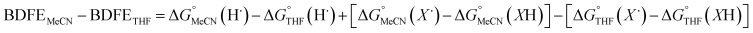


The terms corresponding to 
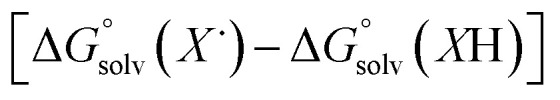
 are primarily dictated by the differences in hydrogen bonding between the radical and reduced version of the organic substrate. Due to the fact that both radical and reduced versions are approximately the same size, other factors that impact solvation can be ignored.^46^ With this in mind, an empirical formula for calculating the difference in hydrogen bonding in aprotic solvents has been developed,^66,67^ shown in eqn (2), where *α*_2_^H^ is the H-bonding acidity constant for the organic reagent, and *β*_2_^H^ is the H-bonding basicity constant for the solvent of interest (*β*_2_^H^(MeCN) = 0.44; *β*_2_^H^(THF) = 0.51). To our knowledge, there are no reports for the *α*_2_^H^ value for DHP; as such we approximate this value using the value reported for diphenylamine (*α*_2_^H^(diphenylamine) = 0.324).^68^2
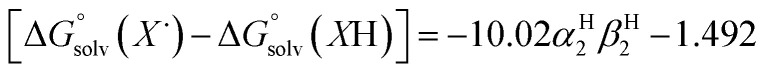


Using these equations, we are able to obtain a calculated BDFE(N–H)_avg_ for DHP in THF (59.2 kcal mol^−1^). This value only differs from the experimentally determined BDFE(N–H)_avg_ for DHP in MeCN by 0.5 kcal mol^−1^.^47^ Accordingly, we are able to confidently conclude that the BDFE(O–H)_avg_ for (calix)V_6_O_5_(OH)_2_ is greater than 59.2 kcal mol^−1^ in THF.

To further benchmark the reactivity of (calix)V_6_O_6_, we explored its behaviour with alternative H-atom transfer reagents that possess stronger E–H bonds. Hydrazobenzene is commonly employed as a reducing reagent (BDFE(N–H)_avg,MeCN_ = 60.9 kcal mol^−1^).^47^ Following the aforementioned strategy, we are able to calculate the average bond strength of the N–H bonds in THF (60.4 kcal mol^−1^). Upon the addition of one equivalent of hydrazobenzene to the oxidized cluster, quantitative formation of the reduced complex (calix)V_6_O_5_(OH_2_) is observed (Fig. S4†). In contrast, addition of 1,4-naphthalenediol (H_2_NQ), a PCET reagent with a slightly stronger E–H bonds (BDFE(O–H)_avg,THF_ = 62.6 kcal mol^−1^ ref. 47 results in a mixture of the oxidized and reduced versions of cluster (*e.g.*(calix)V_6_O_5_(OH_2_)/(calix)V_6_O_6_) and organic substrate (Fig. S5†).

**Scheme 2:**
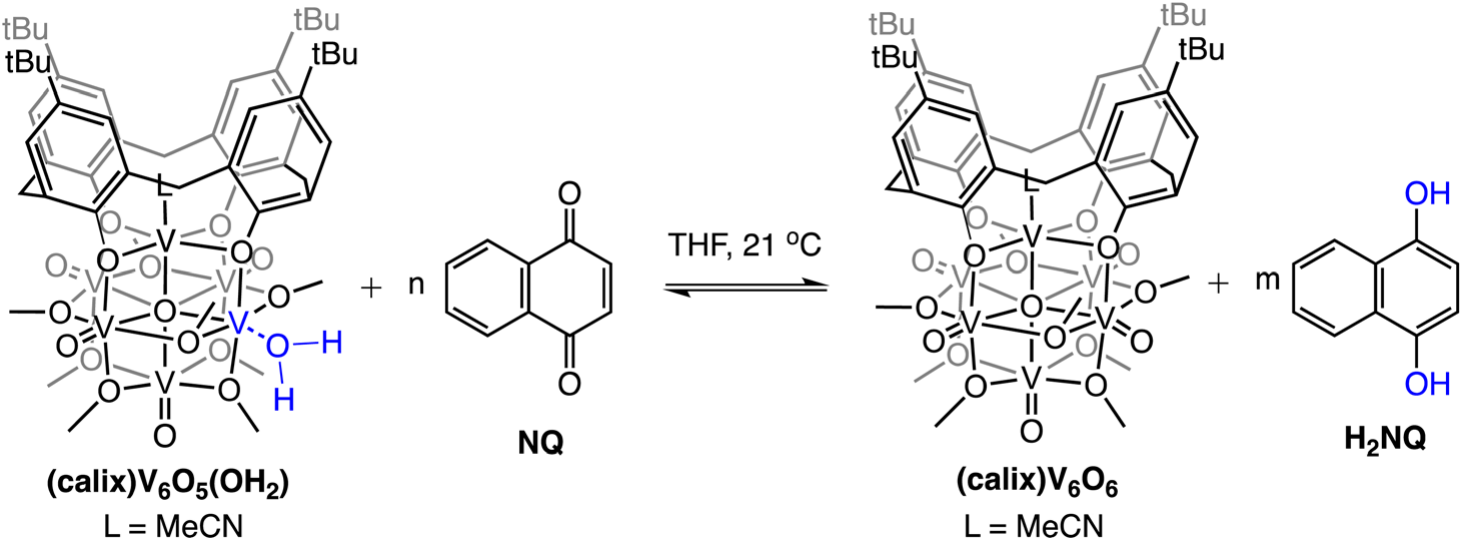
Experiments targeting the determination of BDFE(O–H)_avg_ for (calix)V_6_O_5_(OH_2_). Equilibrium obtained following addition of various concentrations of NQ; adjusted BDFE(O–H) for substrate established using eqn (3). See Experimental section for additional details.

The observed equilibrium between oxidized and reduced versions of the POV-alkoxide cluster following addition of H_2_NQ to (calix)V_6_O_6_ suggests that H-atom transfer is active in both directions (*i.e.*, the cluster is capable of donating and accepting H-atom equivalents). To further probe this finding, we evaluated the ability for H-atom transfer to occur from (calix)V_6_O_5_(OH_2_) to an H-atom accepting reagent, naphthoquinone (NQ; Scheme 2). Upon addition of the organic substrate to the cluster, formation of (calix)V_6_O_6_ is be observed *via*^1^H NMR spectroscopy (Fig. S6†), along with the hydrogenated substrate, H_2_NQ. This observation is significant, as this is the first example in which H-atom transfer is found to occur at the surface of a polyoxometalate cluster from a coordinated aquo ligand. Additionally, this result implies that the O–H bond strengths of the aquo ligand are substantially weakened upon coordination to the reduced vanadium centre in comparison to that of free water (BDFE(O–H)_avg,H2O_ = 108.9 kcal mol^−1^).^47^

Although it is well established that water molecules are capable of associating to surface localized O-atom vacancies, the resultant aquo ligands are typically considered inactive toward the transfer of H-atom equivalents. Instead, aquo ligands either rapidly dissociate from the O-atom vacancy,^69^ or alternatively undergo dissociative adsorption at the surface of the material.^28–31^ To the best of our knowledge, there are no examples in which surface coordinated aquo ligands are capable of facilitating H-atom transfer to small molecule substrates. Our findings constitute the first indication of an alternative pathway by which MO_*x*_ surfaces can hydrogenate small molecule substrates.

**Fig. 3:**
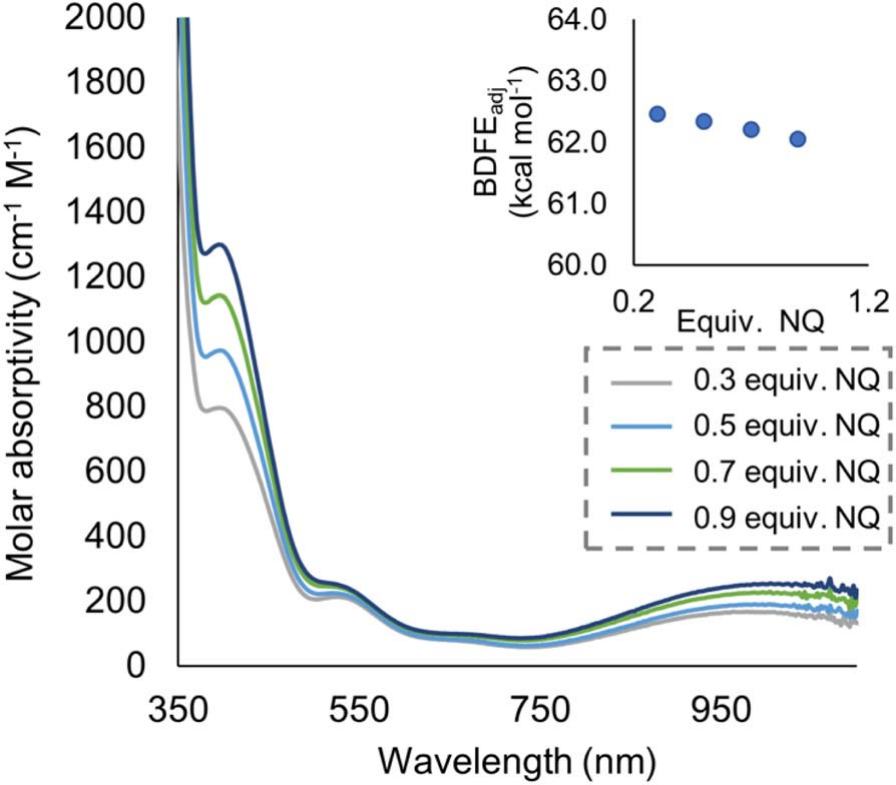
Electronic absorption spectra for samples containing 0.2 mM of the reduced cluster, (calix)V_6_O_5_(OH_2_), and various concentrations of the organic compound, NQ. Concentration of the reduced and oxidized versions of the organic compound were calculated using the original concentration of NQ in solution, and the concentration of reduced cluster remaining in solution after reaching equilibrium. From these results, BDFE(O–H)_avg_ of (calix)V_6_O_5_(OH_2_) is calculated using eqn (3) as shown in inset.

To quantify the free energy of the O–H bonds of the aquo ligand, a series of equilibrium studies were performed (Fig. 3). To begin, we studied the reaction between the reduced cluster, (calix)V_6_O_5_(OH_2_) and NQ (Scheme 2). After 12 hours, no significant change in the product distribution was observed, indicating that the reaction mixture had reached equilibrium. At equilibrium, the affinities of H-atoms for both substrate and cluster surface are identical, allowing for determination of the BDFE(O–H)_avg_ of the surface-bound water molecule (see Experimental section for details).^10^ Obtaining a ratio of [H_2_NQ] to [NQ] at equilibrium allows for determination of the BDFE(O–H)_avg_ for complex (calix)V_6_O_5_(OH_2_) using eqn (3), under the assumption that at equilibrium, the adjusted BDFE(O–H)_avg_ for H_2_NQ will be equal to the BDFE(O–H)_avg_ of (calix)V_6_O_5_(OH_2_).3
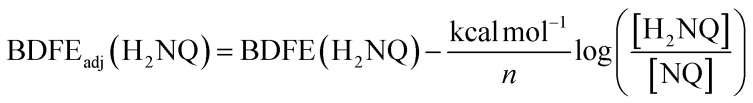
where BDFE_adj_(H_2_NQ) is the average BDFE(N–H) of the reduced organic substrate adjusted for the relative concentrations of the reduced and oxidized organic compound in solution, BDFE(H_2_NQ) is the value reported in THF (62.6 kcal mol^−1^),^47^*n* is the number of H-atom equivalents transferred (*n* = 2 for NQ/H_2_NQ), and [H_2_NQ] and [NQ] are the concentrations of the reduced and oxidized versions of the organic substrate, respectively. Repeating the experiment across a range of added equivalents of NQ (0.3–0.9 equivalents of NQ with respect to (calix)V_6_O_5_(OH_2_), Fig. 3), we are able to measure the BDFE(O–H)_avg_ for (calix)V_6_O_5_(OH)_2_ (62.4 ± 0.2 kcal mol^−1^).

**Fig. 4:**
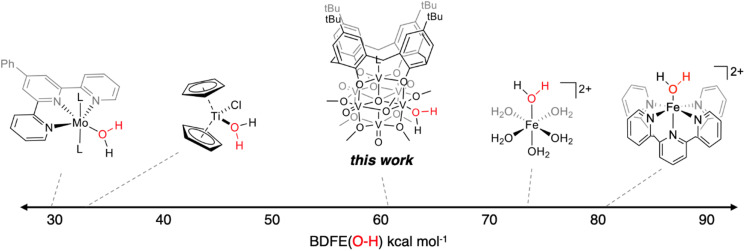
Examples of coordination-induced bond weakening of water in metal aquo complexes.

The experimentally determined BDFE(O–H)_avg_ value for the O–H bonds of the aquo ligand of (calix)V_6_O_5_(OH_2_) is substantially lower than values reported for free H_2_O (BDFE(O–H) = 110.6 kcal mol^−1^, gas phase).^47^ Similar coordination-induced bond weakening has been reported for water ligands bound to reduced metal centres; in all cases, change in BDFE(O–H) is directly correlated to the oxidation potential of the metal centre (Fig. 4). For example, the titanocene(iii) complex reported by Cuevra binds to water, resulting in the formation of a highly reactive O–H bond due to the thermodynamic driving force for oxidation of the Ti(iii) centre.^70,71^ The change in BDFE(O–H) of water reported here, following coordination to a V(iii) center embedded within a multimetallic MO_*x*_ assembly, is smaller than that described for Cp_2_TiCl(OH_2_) (ΔBDFE(O–H) = 46.5 kcal mol^−1^ for V(iii) *vs.* ΔBDFE(O–H) = 58.7 kcal mol^−1^ for Ti(iii)), consistent with the modest oxidation potential of the parent cluster (*E*_1/2_ = −0.477 V *vs.* Fc^+/0^ in THF, Fig. S7†).

The BDFE(O–H)_avg_ obtained for the reduced cluster, (calix)V_6_O_5_(OH_2_), closely resembles values previously reported by our group for reduced polyoxovanadate cluster containing a similar Lindqvist structure.^12^ However, in our previous work, it was determined that reactivity occurs exclusively at bridging oxide ligands, whereas in this work, reactivity is limited to the terminal vanadyl site. The similarity between these two values is surprising, given the differences in the coordination geometry of the active O-atom. Computational analysis into H-atom uptake at ceria oxide nanoparticles reveals substantial differences in the energy required for hydrogenation to occur at the surface of the compound based on whether the active O-atom bridges two metal centres (M–O–M) or a terminal ligand (M=O).^72^ However, our experiments suggest an apparent insensitivity to the identity of the active site on the bond strength of O–H bonds at reduced polyoxovanadate surfaces, contradicting these theoretical findings. This result has significant bearing on the design of catalytically active surfaces, and warrants further consideration by the community.

### Mechanistic analysis of PCET from (calix)V_6_O_5_(OH_2_)

Establishing of the BDFE(O–H)_avg_ of (calix)V_6_O_5_(OH_2_) allows for the prediction of the thermochemical driving force for PCET from the cluster surface to a given substrate. However, this information alone provides an incomplete description of charge transfer reactions. While thermodynamics are a critical factor to consider when designing systems for the mediation of PCET reactions, it is important to account for kinetic influences on the reaction. For instance, PCET reactions that proceed *via* a stepwise mechanism, with either the electron or proton transferring initially, result in the formation of charged intermediates. These pathways often require overcoming large activation barriers, and as a consequence, may experience sluggish reaction kinetics. However, the transfer of both electron and proton together in a single kinetic step, known as concerted proton–electron transfer (CPET), can, in some circumstances, avoid the formation of these high energy intermediates.

**Scheme 3:**
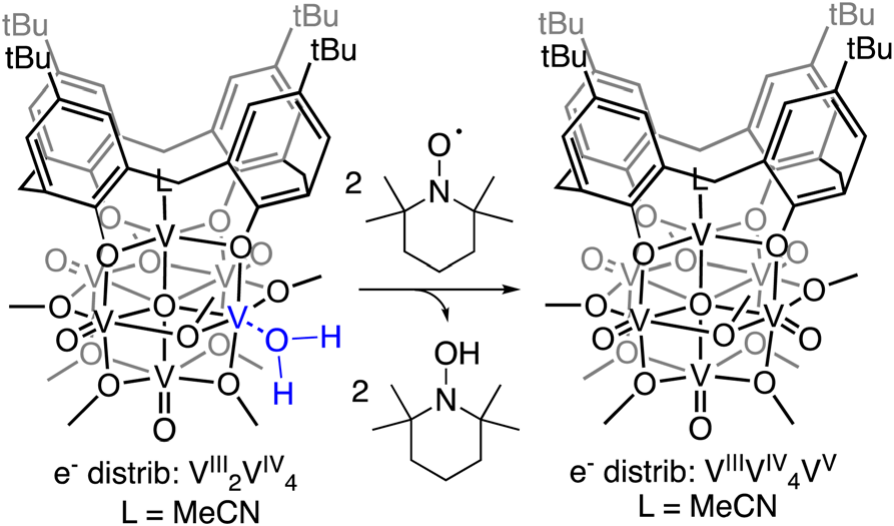
H-atom transfer reactivity from (calix)V_6_O_5_(OH_2_) to TEMPO.

To probe the mechanism of H-atom transfer from the coordinated water molecule at the surface of the reduced vanadium oxide cluster, we performed a series of pseudo-first order reaction analyses. We opted to investigate H-atom transfer between the reduced cluster, (calix)V_6_O_5_(OH_2_), and an excess of the substrate, TEMPO (2,2,6,6-tetramethylpiperidin-1-oxyl; 20–120 equivalents; Scheme 3). TEMPO is an ideal substrate, as it allows for the interrogation of single *vs.* multi-HAT in the rate determining step of the reaction. This substrate is also exceptionally difficult to reduce in the absence of protons in THF (*E*_c_ = −2.56 V *vs.* Fc^+/0^, Fig. S8†), eliminating the possibility of a stepwise ET-PT pathway.

**Fig. 5:**
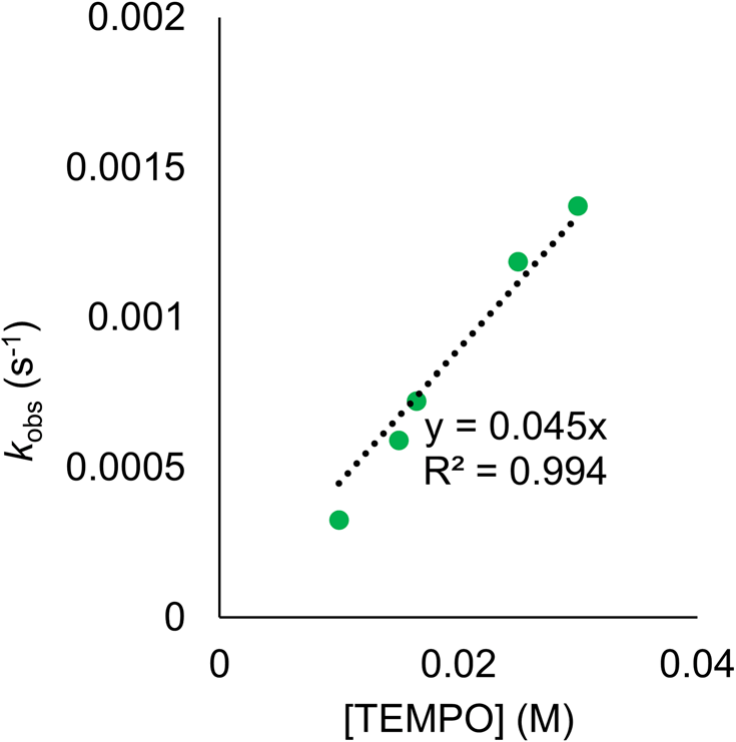
Plot of *k*_obs_ against the concentration of TEMPO initially in solution for the reaction between (calix)V_6_O_5_(OH_2_) and TEMPO. Each sample contains 0.5 mM of (calix)V_6_O_5_(OH_2_) in THF and run at 25 °C. The linear trend of these results indicates the reaction is first order with respect to TEMPO. The second order rate constant, *k*_PCET_ can be determined from the slope of the best fit line with a set *y*-intercept of 0, where ½ of the slope is equal to *k*_PCET_ due to the presence of two chemically equivalent H-atoms at the surface of the reduced cluster.

Rate constants were determined by monitoring the change in the electronic absorption spectrum for (calix)V_6_O_5_(OH_2_) over time. The growth of the low intensity absorption feature at 900 nm is indicative of the formation of the oxidized cluster, (calix)V_6_O_6_ (*vide supra*). Measuring the change in absorbance as a function of time results in a kinetic trace as seen in Fig. S9.† Extraction of the pseudo first-order rate constant (*k*_obs_) is performed by determining the slope of the plot ln((*A*_*t*_ − *A*_inf_)/(*A*_0_ − *A*_inf_)) *vs.* time, where *A*_*t*_ is absorbance at time = *t*, A_inf_ is the absorbance at the end of the reaction, and *A*_0_ is the initial absorbance (Fig. S10;† see Experimental section for more details). Plotting *k*_obs_ against the concentration of TEMPO reveals a linear correlation (Fig. 5), suggesting the reaction is first order with respect to TEMPO. The linear relationship between time and the natural log of the concentration of the oxidized cluster noted in Fig. S10† indicates that the reaction is also first order with respect to cluster, translating to an overall second order rate law for the reaction. This suggests that the rate limiting step of the reaction involves the transfer of only one H-atom from the aquo ligand to a TEMPO molecule. The second order rate constant for PCET at (calix)V_6_O_5_(OH_2_) (*k*_PCET_) can be found from the slope in Fig. 5, where *k*_PCET_ is equal to ½ × slope of the best fit line to account for the two chemically identical O–H bonds of the aquo ligand, resulting in a *k*_PCET_ value of 2.3 × 10^−2^ M^−1^ s^−1^.

Due to the fact that kinetic analysis of PCET between reduced polyoxometalates and organic substrates is a relatively new area, there are few examples available for comparison of the observed rate constant. One such study from our group determined the rate constant for the reduction of TEMPO by the reduced POV-alkoxide cluster, [V_6_O_11_(OH)_2_(TRIOL^NO_2_^)_2_]^−2^. Interestingly, the overall rate constant for this previous example is two orders of magnitude larger than the value reported in this work, despite the fact that H-atom transfer from (calix)V_6_O_5_(OH_2_) is thermodynamically preferred. The observed discrepancy in the expected rates of reaction can be attributed to a kinetic solvent effect (KSE). The previous study from our group was performed in acetonitrile and observed no significant interaction between the reduced cluster and solvent. Conversely, structural analysis of (calix)V_6_O_5_(OH_2_) suggests strong interactions between the aquo ligand and THF. This H-bonding network must be disrupted prior to H-atom transfer. KSE has been shown to impart significant kinetic barriers for PCET as a result of increases in the activation barrier required to reach the transition state of the reaction and is likely the cause of the relatively sluggish reaction kinetics observed here.^73^

Next, kinetic isotope effect (KIE) studies were performed; formation of the deuterated derivative of the reduced cluster, (calix)V_6_O_5_(OD_2_), was achieved *via* reduction of (calix)V_6_O_6_ using the deuterated compound, *d*_2_-DHP (see Experimental section for details, Fig. S11–S13†). Repeating the pseudo-first order reaction experiments using the deuterated cluster, (calix)V_6_O_5_(OD_2_), reveals a decrease in the overall rate of reaction (Fig. S14†). Plotting the *k*_obs_ values against the concentration of excess TEMPO results in a *k*_PCET_ value of 1.4 × 10^−2^ M^−1^ s^−1^. Comparison rate constants of H-atom transfer from the protonated and deuterated clusters results in a KIE value of 1.61. While a KIE value > 1 suggests a rate determining step that includes the cleavage of the O–H bond at the cluster, this value is not substantial enough to conclusively assign a reaction mechanism.^74–77^

**Fig. 6:**
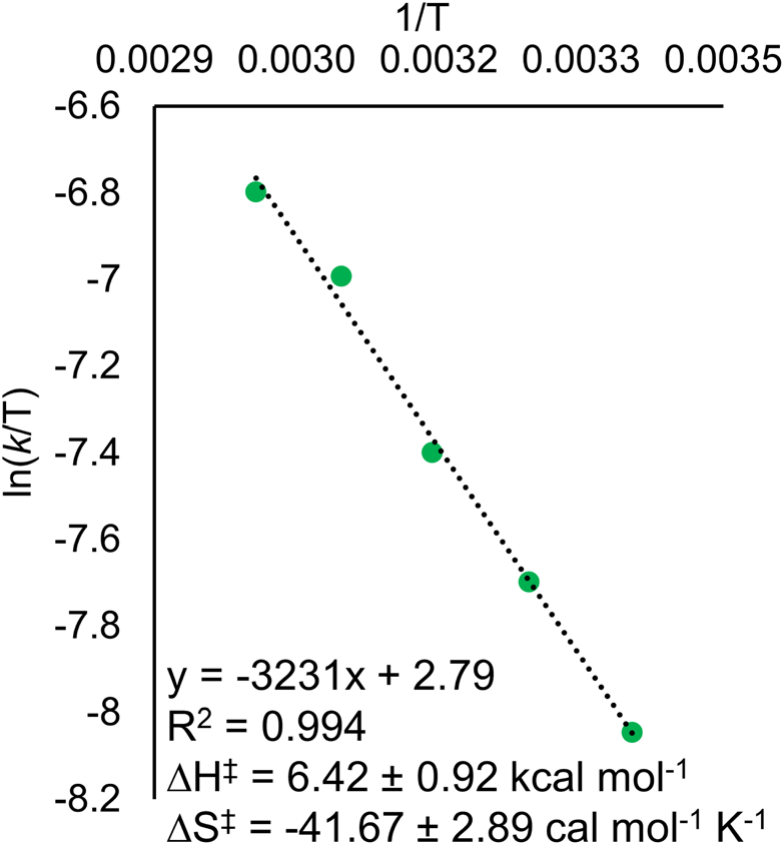
Plot of *k*_obs_ against the concentration of TEMPO initially in solution for the Eyring plot for the reaction between (calix)V_6_O_5_(OH_2_) and TEMPO. Reactions were run in pseudo-first order conditions, with the concentration of the cluster at 0.5 mM and the concentration of TEMPO at 30 mM across each experiment. The temperature of the reaction was varied between 25 – 65 °C. Rate constants for the *y*-axis were found by dividing the observed rate constant by the concentration of TEMPO to obtain the rate constant, *k*_PCET_.

Eyring analysis of the H-atom transfer process was performed to unambiguously distinguish between CPET and PT/ET pathways. We measured *k*_obs_ under pseudo-first order reaction conditions across a range of temperatures (25–65 °C). From this information, determination of activation parameters for H-atom transfer from (calix)V_6_O_5_(OH_2_) to TEMPO is possible (Δ*H*^‡^ = 6.1 ± 0.9 kcal mol^−1^; Δ*S*^‡^ = −41.7 ± 2.9 cal mol^−1^ K^−1^; Δ*G*^‡^ = 18.8 ± 1.8 kcal mol^−1^; Fig. 6). The Δ*H*^‡^ value is comparable to values obtained previously for a concerted PCET reaction at MO_*x*_ compounds.^12,14,78,79^ The relatively small magnitude of Δ*H*^‡^ can be explained by the fact that (calix)V_6_O_5_(OH_2_) is likely able to form a stable hydrogen bond to TEMPO, suggesting the reaction requires relatively little enthalpic energy to reach the activated transition state.^80^

The large negative value for Δ*S*^‡^ suggests a highly ordered intermediate is formed in the transition state of the rate determining step of H-atom transfer. This finding is consistent with a TEMPO molecule forming a H-bonding interaction with the surface aquo ligand of (calix)V_6_O_5_(OH_2_) prior to the formal transfer of the H-atom. Previous reports of MO_*x*_ compounds performing H-atom transfer reactions *via* CPET mechanisms found similarly negative Δ*S*^‡^ values. This can be explained by the fact that CPET is inherently an inner sphere process; formation hydrogen bonds between the H-atom acceptor and donor are required for the reaction to occur.^78,79^

## Conclusion

Here, we have described the synthesis and isolation of a reduced hexavanadate cluster, (calix)V_6_O_5_(OH_2_). While previous studies from our laboratory have demonstrated the predilection of reduced polyoxovanadate clusters to form bridging hydroxide ligands, this work reports the first instance of the formation of a stable aquo ligand at the surface of the cluster. Notably, H-atom transfer reactivity is observed from (calix)V_6_O_5_(OH_2_), indicating that the cluster is capable of performing proton coupled electron transfer reactions using the H-atoms of water. Experimental analysis results in the direct measurement of the BDFE(O–H)_avg_ of the aquo ligand (62.4 ± 0.2 kcal mol^−1^). Mechanistic analysis reveals that H-atom transfer reaction is likely occurring through a CPET pathway.

The experimentally determined BDFE(O–H)_avg_ of the reduced cluster, (calix)V_6_O_5_(OH_2_) (62.4 ± 0.2 kcal mol^−1^), demonstrates that coordination of water to an O-atom defect site results in a substantial increase in the thermodynamic driving force of H-atom transfer from water. Indeed, the BDFE(O–H) of water decreases ∼50 kcal mol^−1^ upon coordination to the cluster surface (BDFE(H_2_O)_avg_ = 108.9 kcal mol^−1^).^47^ This phenomenon, commonly referred to as coordination induced bond weakening, has been observed previously in mononuclear transition metal complexes. Significantly, the BDFE(O–H)_avg_(calix)V_6_O_5_(OH_2_) exists in a “sweet spot”, weak enough to enable the cluster to act as a potent H-atom donor to a variety of small molecule substrates, while avoiding H_2_ formation. This is in contrast to the BDFE(O–H) values reported for early transition metal aquo species, which have been reported at values below the point at which the formation of H_2_ is thermodynamically favourable (BDFE(H_2_) ≈ 52 kcal mol^−1^).^81,82^ Indeed, this finding indicates that reduced polyoxovanadate-alkoxide clusters are promising candidates for use as catalysts for hydrogenation reactions.

The structural and electronic similarities of the POV-alkoxide cluster to bulk MO_*x*_ allow for valuable insight into surface reactivity of these materials. Although many studies have demonstrated the ability for MO_*x*_ to facilitate PCET,^10,83–86^ identification of individual active sites and elucidation of precise reaction mechanisms is challenging, in part due to constraints of spectroscopic techniques available for heterogeneous systems. Furthermore, theoretical analysis of PCET at MO_*x*_ surfaces often localizes reactivity to bridging hydroxide ligands, disregarding the possibility of the participation of aquo moieties in H-atom transfer reactions. In light of this, the results reported here provide insight into a novel pathway of H-atom transfer at the surface of redox-active metal oxides, whereupon coordination of water to an O-atom defect site, the O–H bonds of the substrate are activated. Coordination-induced bond weakening enables hydrogenation of the desired substrate. The ability to derive H-atom equivalents from water is a significant finding, as commonly H_2_ used in these molecular transformations is obtained through the combustion of fossil fuels. When taken together, this work provides valuable insight into novel mechanisms of H-atom transfer at the surface MO_*x*_ compounds, revealing design criteria for MO_*x*_-derived hydrogenation catalysts.

## Experimental

### General considerations

All manipulations were carried out in the absence of water and oxygen using standard Schlenk techniques or in a UniLab MBraun inert atmosphere dry-box under a dinitrogen atmosphere. All glassware was oven-dried for a minimum of 4 h and cooled in an evacuated antechamber prior to use in the dry-box. Solvents were dried and deoxygenated on a glass contour system (Pure Process Technology, LLC) and stored over 3 Å molecular sieves purchased from Fisher Scientific and activated prior to use. TEMPO was purchased from Sigma-Aldrich and used as received. Hydrazobenzene was purchased from TCI and used as received. The POV-alkoxide cluster, (calix)V_6_O_6_, was prepared according to previously reported procedure.^44^ 5,10-dihydrophenazine^87^ and 5,10-dideuterophenazine,^13^ were generated following literature precedent. 1,4-Naphthalenediol was formed following similar procedures for the formation of 1,4-hydroquinone, where substitution of the respective organic substrate resulted in the formation of the desired reduced organic compound.^45^

^1^H NMR spectra were recorded at 400 MHz or 500 MHz on a Bruker DPX-400 or Bruker DPX-500 spectrometer, locked on the signal of deuterated solvents. All chemical shifts were reported relative to the peak of the residual H signal in deuterated solvents. CDCl_3_ and THF-*d*_8_ was purchased from Cambridge Isotope Laboratories, degassed by three freeze–pump–thaw cycles, and stored over fully activated 3 Å molecular sieves. Infrared (FT-IR, ATR) spectra were recorded on a Shimadzu IRAffinity-1 Fourier transform infrared spectrophotometer and are reported in wavenumbers (cm^−1^). Electronic absorption spectra were recorded at room temperature in anhydrous THF in a sealed 1 cm quartz cuvette with an Agilent Cary 60 UV-vis spectrophotometer.

A single crystal of (calix)V_6_O_5_(MeCN)(OH_2_)(OMe)_8_ ((calix)V_6_O_5_(OH_2_)) was placed onto a thin glass optical fiber or a nylon loop and mounted on a Rigaku XtaLAB Synergy-S Dualflex diffractometer equipped with a HyPix-6000HE HPC area detector for data collection at 173.00(10) K. A preliminary set of cell constants and an orientation matrix were calculated from a small sampling of reflections.^88^ A short pre-experiment was run, from which an optimal data collection strategy was determined. The full data collection was carried out using a PhotonJet (Cu) X-ray source with frame times of 3.37 and 13.49 seconds and a detector distance of 34.0 mm. After the intensity data were corrected for absorption, the final cell constants were calculated from the xyz centroids of 17 304 strong reflections from the actual data collection after integration.^88^ The structure was solved using SHELXT^89^ and refined using SHELXL.^90^ The space group *Pbcm* was determined based on systematic absences and intensity statistics. Most or all non H-atoms were assigned from the solution. Full-matrix least squares/difference Fourier cycles were performed which located any remaining non H-atoms. All non H-atoms were refined with anisotropic displacement parameters. The O–H H-atoms (one unique) on atom O4 were found from the difference Fourier map and refined freely. All other H-atoms were placed in ideal positions and refined as riding atoms with relative isotropic displacement parameters.

### Synthesis of (calix)V_6_O_5_(MeCN)(OH_2_)(OMe)_8_, (calix)V_6_O_5_(OH_2_)

A 20 mL scintillation vial was charged with (calix)V_6_O_6_ (0.026 g, 0.019 mmol) and 6 mL THF. Dihydrophenazine (0.005 g, 0.025 mmol) was added as a solid with stirring. The reaction was stirred at room temperature for 1 h, whereupon a colour change from brown to red was observed. The crude solid dried under reduced pressure and was washed with pentane (3 mL × 3) to remove the by-product, phenazine. The red solid was extracted in THF and was dried *in vacuo*. Recrystallization of the product occurred through slow diffusion of pentane into THF resulting in the isolation of red, starburst-shaped crystals of the product, (calix)V_6_O_5_(OH_2_), suitable for single crystal X-ray diffraction (0.011 g, 0.008 mmol, 57%). ^1^H NMR (400 MHz, CDCl_3_): (*δ* = 28.74, 26.47, 8.48, 7.89, 6.86, 1.21, −3.79, −5.11, −11.64). FT-IR (ATR, cm^−1^): 1042 (O_b_–CH_3_), 982 (V=O_t_). UV-vis (THF, 21 °C): 900 nm (*ε* = 1163 M^−1^ cm^−1^), 521 nm (*ε* = 155 M^−1^ cm^−1^), 414 nm (*ε* = 234 M^−1^ cm^−1^), 400 nm (sh, *ε* = 100 M^−1^ cm^−1^). Elemental analysis: calc. for C_54_H_76_O_20_V_6_·MeOH·CHCl_3_ (*M*_W_ = 1480.23): C, 48.12; H, 6.06. Found: C, 47.8; H, 5.9; N, 0.18.

### Synthesis of (calix)V_6_O_5_(MeCN)(OD_2_)(OMe)_8_, (calix)V_6_O_5_(OD_2_)

A 20 mL scintillation vial was charged with (calix)V_6_O_6_ (0.035 g, 0.026 mmol) and 6 mL THF. Deuterated dihydrophenazine (0.005 g, 0.028 mmol) was added as a solid with stirring. The reaction was stirred at 25 °C for 2 hours, whereupon a color change from brown to red was observed. The crude solid dried under reduced pressure and was washed with pentane (3 mL × 3) to remove phenazine, filtering over Celite. The red solid was extracted in THF and was dried *in vacuo* which resulted in the formation of (calix)V_6_O_5_(OD_2_) (0.016 g, 0.012 mmol, 45%). To confirm deuterium transfer to the cluster, 2 eq of TEMPO was added to the cluster in THF and ^2^H NMR was taken, and TEMPO-D was observed (1.93 ppm, s). ^1^H NMR (400 MHz, CDCl_3_): (*δ* = 28.90, 26.65, 8.45, 7.91, 1.15, −4.49, −10.98).

### General procedure for determining BDFE(O–H) of V_6_O_5_(OH_2_) through equilibrium experiments

In an N_2_-filled glove box, 1 mL of a 0.598 mM stock solution of (calix)V_6_O_5_(OH_2_) in THF and 2 mL of THF are added to a long-necked UV-vis cuvette. The cuvette is capped with a rubber septum and removed from the glove box, where a control electronic absorption spectrum is collected. In the glovebox, in a separate cuvette, 1 mL of the (calix)V_6_O_5_(OH_2_) stock solution is added, along with 1.9 mL of THF, along with 0.1 mL of a stock solution containing 0.62 mM naphthoquinone in THF. The sample was shaken several times to ensure homogeneity and allowed to sit at 25 °C for 12 hours, whereupon an electronic absorption spectrum was collected. This procedure was repeated for other equivalents of naphthoquinone. The extent of the reaction is determined through the absorbance measured at 900 nm, where the intervalence charge transfer band of (calix)V_6_O_6_ allows for the determination of the concentration of each cluster. The concentration of 1,4-naphthalenediol and naphthoquinone are established by relative amounts of reduced and oxidized cluster present in solution, under the assumption that H-atom transfer occurs solely from the cluster to substrate (*e.g.* for each oxidized cluster formed, we assume the reduction of one molecule of naphthoquinone). Calculating the BDFE(O–H) of the reduced cluster in solution can then be performed through methods adapted from the Mayer group,^10^ using eqn (4).4
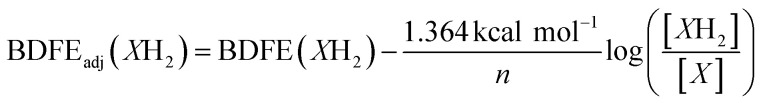
where BDFE_adj_(*X*H_2_) is the adjusted BDFE of the organic substrate based on where the equilibrium of the system lies, BDFE(*X*H_2_) is the reported BDFE(O–H) of the reduced version of naphthoquinone (62.6 kcal mol^−1^ in THF^47^), *R* is the gas constant, *T* is the temperature, *n* is the number of H-atoms transferred to one equivalent of naphthoquinone (*n* = 2 in these experiments, as naphthoquinone can accept two H-atom equivalents), and [*X*H_*n*_] and [*X*] are the measured concentrations of reduced and oxidized versions of the respective substrate in solution at equilibrium.

### General procedure for measuring kinetics of PCET at POV clusters through electronic absorption

Each sample was monitored using electronic absorption spectroscopy, where the absorbance at 900 nm is used to determine the concentration of the oxidized cluster, (calix)V_6_O_6_, present in solution. All experiments are performed in 3 mL of THF at the desired temperature. An initial sample of 0.5 mM (calix)V_6_O_5_(OH_2_) was prepared in THF in an air free quartz cuvette. A sample of stock solution containing TEMPO was collected in an air-free syringe and both cuvette and syringe are removed from the glove box. The cuvette is inserted into the UV-vis spectrophotometer and allowed to equilibrate to the desired temperature. Data collection begins as soon as the organic substrate is rapidly injected into the cuvette. Once the sample reaches equilibrium, and the absorbance of the sample is no longer changing, the data collection is stopped. The sample is then disposed of in the waste, and the cuvette is washed and dried in an oven at 125 °C for 2 hours before being brought back into the glove box. The absorbance of the sample was plotted *versus* time, and k_obs_ extrapolated from a linear trend line of time *vs.* ln((*A* − A_inf_)/*A*_0_ − *A*_inf_), where *A*_*t*_ is absorbance at time = *t*, *A*_inf_ is the absorbance of the sample once equilibrium is reached, *A*_0_ is the initial absorbance of the sample, *k*_obs_ is the observed rate constant in pseudo-first order reaction conditions, and *t* is time in seconds.

The slope of the line of the plot is equal to *k*_obs_ which can then be found for each of the different concentrations of TEMPO. *k*_PCET_ can then be found plotting a line of *k*_obs_*vs.* [TEMPO] from ½ of the slope to account for the two chemically equivalent hydrogens on the aquo moiety.

### General procedure for determining activation energy for the oxidation of (calix)V_6_O_5_(OH_2_) by HAT

Activation parameters were determined using kinetic data obtained by measuring the initial rate of formation of the product cluster, (calix)V_6_O_6_, over a range of temperatures (25 → 65 °C). Pseudo first-order reaction conditions were used to simplify finding the observed rate constants for each temperature, where the concentration of TEMPO was held in excess over the cluster. Rate constants were found by dividing the observed rate constant by the concentration of TEMPO to obtain the rate constant, *k*. From the results collected in the variable temperature experiments, the activation parameters are able to be established using the linear form of the Eyring–Polanyi equation shown in eqn (5).5
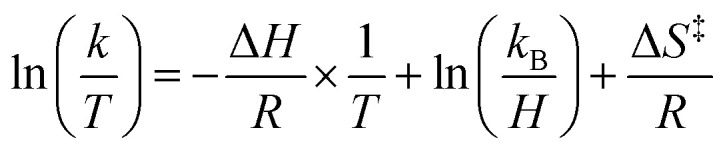

6 Δ*G*^‡^ = Δ*H*^‡^ − *T*Δ*S*^‡^

where *T* is the temperature, Δ*H*^‡^ is the enthalpy of activation, Δ*S*^‡^ is the entropy of activation, *R* is the universal gas constant (*R* = 1.987 × 10^−3^ kcal K^−1^ mol^−1^), *k*_B_ is the Boltzmann constant, and *h* is Planck's constant. Plotting ln(*k*/*T*) *vs.* 1/*T* gives a plot with a linear best-fit line, from which the enthalpy of activation can be found by slope = −Δ*H*^‡^/*R*. In addition, the entropy of activation can be found from the *y*-intercept, where *y*-intercept = ln(*k*_B_/*h*) + Δ*S*^‡^/*R*. From these parameters, the activation free energy can be determined at the desired temperature using eqn (6). All values are reported at 95% confidence interval by linear regression using excel.

## Data availability

Crystallographic data for compound (calix)V_6_O_5_(OH_2_) has been deposited in the Cambridge Crystallographic Data Centre (CCDC) under accession number 2203190 and can be obtained online. All other datasets for this article have been uploaded as part of the ESI.†

## Author contributions

S. E. C., A. A. F., and E. M. M. conceived and planned the experiments. S. E. C., A. A. F., and M. R. B. performed the synthesis of complexes and collected all experimental data, expect those involving X-ray crystallography. W. W. B. collected single crystal X-ray diffraction data and solved the crystal structures. All authors contributed to the writing of the manuscript.

## Conflicts of interest

There are no conflicts to declare.

## Supplementary Material

SC-013-D2SC04843D-s001

SC-013-D2SC04843D-s002

## References

[cit1] Xue Y., Sun S., Wang Q., Dong Z., Liu Z. (2018). J. Mater. Chem. A.

[cit2] Xu T., Liang J., Li S., Xu Z., Yue L., Li T., Luo Y., Liu Q., Shi X., Asiri A. M., Yang C., Sun X. (2021). Small Sci..

[cit3] Napporn T. , Holade Y. and Korotcenkov G., Metal Oxide-Based Nanostructured Electrocatalysts for Fuel Cells, Electrolyzers, and Metal–Air Batteries, Elsevier Science, 2021

[cit4] Zhang X., Ye L., Xie K. (2022). Energy Fuels.

[cit5] Subotić V. and Napporn T. W., in Metal Oxide-Based Nanostructured Electrocatalysts for Fuel Cells, Electrolyzers, and Metal–air Batteries, ed. T. W. Napporn and Y. Holade, Elsevier, 2021, pp. 213–234, 10.1016/B978-0-12-818496-7.00003-5

[cit6] Hu A., Jiang Z., Kuai C., McGuigan S., Nordlund D., Liu Y., Lin F. (2020). J. Mater. Chem. A.

[cit7] Prins R. (2012). Chem. Rev..

[cit8] Fleischmann S., Mitchell J. B., Wang R., Zhan C., Jiang D.-e., Presser V., Augustyn V. (2020). Chem. Rev..

[cit9] Norby T., Widerøe M., Glöckner R., Larring Y. (2004). Dalton Trans..

[cit10] Agarwal R. G., Kim H.-J., Mayer J. M. (2021). J. Am. Chem. Soc..

[cit11] Amtawong J., Nguyen A. I., Tilley T. D. (2022). J. Am. Chem. Soc..

[cit12] Fertig A. A., Brennessel W. W., McKone J. R., Matson E. M. (2021). J. Am. Chem. Soc..

[cit13] Schreiber E., Fertig A. A., Brennessel W. W., Matson E. M. (2022). J. Am. Chem. Soc..

[cit14] Amtawong J., Skjelstad B. B., Balcells D., Tilley T. D. (2020). Inorg. Chem..

[cit15] Amtawong J., Balcells D., Wilcoxen J., Handford R. C., Biggins N., Nguyen A. I., Britt R. D., Tilley T. D. (2019). J. Am. Chem. Soc..

[cit16] Song F., Moré R., Schilling M., Smolentsev G., Azzaroli N., Fox T., Luber S., Patzke G. R. (2017). J. Am. Chem. Soc..

[cit17] Pope M. T., Papaconstantinou E. (1967). Inorg. Chem..

[cit18] Shanmugaprabha T., Selvakumar K., Vairalakshmi M., Rajasekaran K., Sami P. (2015). Transition Met. Chem..

[cit19] Bezdek M. J., Guo S., Chirik P. J. (2016). Science.

[cit20] Richens D. T. , The Chemistry of Aqua Ions: Synthesis, Structure and Reactivity: ATour Through the Periodic Table of the Elements, Wiley, 1997

[cit21] Goldsmith C. R., Stack T. D. P. (2006). Inorg. Chem..

[cit22] Cuerva J. M., Campaña A. G., Justicia J., Rosales A., Oller-López J. L., Robles R., Cárdenas D. J., Buñuel E., Oltra J. E. (2006). Angew. Chem., Int. Ed..

[cit23] Dhar D., Tolman W. B. (2015). J. Am. Chem. Soc..

[cit24] Moyer B. A., Meyer T. J. (1981). Inorg. Chem..

[cit25] Goldsmith C. R., Cole A. P., Stack T. D. P. (2005). J. Am. Chem. Soc..

[cit26] Dhar D., Yee G. M., Spaeth A. D., Boyce D. W., Zhang H., Dereli B., Cramer C. J., Tolman W. B. (2016). J. Am. Chem. Soc..

[cit27] VanNatta P. E., Ramirez D. A., Velarde A. R., Ali G., Kieber-Emmons M. T. (2020). J. Am. Chem. Soc..

[cit28] Jossou E., Malakkal L., Dzade N. Y., Claisse A., Szpunar B., Szpunar J. (2019). J. Phys. Chem. C.

[cit29] Litke A., Hensen E. J. M., Hofmann J. P. (2017). J. Phys. Chem. C.

[cit30] Nadeem I. M., Treacy J. P. W., Selcuk S., Torrelles X., Hussain H., Wilson A., Grinter D. C., Cabailh G., Bikondoa O., Nicklin C., Selloni A., Zegenhagen J., Lindsay R., Thornton G. (2018). J. Phys. Chem. Lett..

[cit31] Tilocca A., Selloni A. (2004). J. Phys. Chem. B.

[cit32] Piepgrass K., Pope M. T. (1987). J. Am. Chem. Soc..

[cit33] Jeannin Y., Launay J. P., Sedjadi M. A. S. (1980). Inorg. Chem..

[cit34] Kazansky L. P., Launay J. P. (1977). Chem. Phys. Lett..

[cit35] Launay J. P. (1976). J. Inorg. Nucl. Chem..

[cit36] Piepgrass K., Pope M. T. (1989). J. Am. Chem. Soc..

[cit37] Petel B. E., Matson E. M. (2020). Chem. Commun..

[cit38] Petel B. E., Matson E. M. (2021). Inorg. Chem..

[cit39] Petel B. E., Meyer R. L., Brennessel W. W., Matson E. M. (2019). Chem. Sci..

[cit40] Petel B. E., Brennessel W. W., Matson E. M. (2018). J. Am. Chem. Soc..

[cit41] Petel B. E., Fertig A. A., Maiola M. L., Brennessel W. W., Matson E. M. (2019). Inorg. Chem..

[cit42] Petel B. E., Matson E. M. (2020). Chem. Commun..

[cit43] Schreiber E., Petel B. E., Matson E. M. (2020). J. Am. Chem. Soc..

[cit44] Meyer R. L., Miró P., Brennessel W. W., Matson E. M. (2021). Inorg. Chem..

[cit45] Wise C. F., Agarwal R. G., Mayer J. M. (2020). J. Am. Chem. Soc..

[cit46] Warren Jeffrey J., Mayer James M. (2010). Proc. Natl. Acad. Sci. U. S. A..

[cit47] Agarwal R. G., Coste S. C., Groff B. D., Heuer A. M., Noh H., Parada G. A., Wise C. F., Nichols E. M., Warren J. J., Mayer J. M. (2022). Chem. Rev..

[cit48] Petel B. E., Meyer R. L., Maiola M. L., Brennessel W. W., Müller A. M., Matson E. M. (2020). J. Am. Chem. Soc..

[cit49] Fertig A. A., Cooney S. E., Meyer R. L., Brennessel W. W., Matson E. M. (2022). Chem. Commun..

[cit50] VanGelder L. E., Kosswattaarachchi A. M., Forrestel P. L., Cook T. R., Matson E. M. (2018). Chem. Sci..

[cit51] VanGelder L. E., Forrestel P. L., Brennessel W. W., Matson E. M. (2018). Chem. Commun..

[cit52] VanGelder L. E., Brennessel W. W., Matson E. M. (2018). Dalton Trans..

[cit53] Daniel C., Hartl H. (2009). J. Am. Chem. Soc..

[cit54] Zabierowski P., Radoń M., Szklarzewicz J., Nitek W. (2014). Inorg. Chem. Commun..

[cit55] Li F., VanGelder L. E., Brennessel W. W., Matson E. M. (2016). Inorg. Chem..

[cit56] Jeffrey G. A. , An Introduction to Hydrogen Bonding, Oxford University Press, 1997

[cit57] Ren D. X., Xing N., Shan H., Chen C., Cao Y. Z., Xing Y. H. (2013). Dalton Trans..

[cit58] Grey I. E., Madsen I. C., Sirat K., Smith P. W. (1985). Acta Crystallogr., Sect. C: Cryst. Struct. Commun..

[cit59] Tregenna-Piggott P. L. W., Carver G. (2004). Inorg. Chem..

[cit60] Sobota P., Fritsky I. O., Ejfler J., Szafert S., Głowiak T. (1996). Polyhedron.

[cit61] Miyoshi K., Wang J., Mizuta T. (1995). Inorg. Chim. Acta.

[cit62] Shimoi M., Saito Y., Ogino H. (1991). Bull. Chem. Soc. Jpn..

[cit63] Shimoi M., Saito Y., Ogino H. (1989). Chem. Lett..

[cit64] Shimoi M., Miyamoto S., Ogino H. (1991). Bull. Chem. Soc. Jpn..

[cit65] Brunner E. (1985). J. Chem. Eng. Data.

[cit66] Abraham M. H., Grellier P. L., Prior D. V., Taft R. W., Morris J. J., Taylor P. J., Laurence C., Berthelot M., Doherty R. M. (1988). et al.. J. Am. Chem. Soc..

[cit67] Litwinienko G., Ingold K. U. (2007). Acc. Chem. Res..

[cit68] Abraham M. H., Grellier P. L., Prior D. V., Duce P. P., Morris J. J., Taylor P. J. (1989). J. Chem. Soc., Perkin Trans. 2.

[cit69] Bernal S., Calvino J. J., Cifredo G. A., Gatica J. M., Omil J. A. P., Pintado J. M. (1993). J. Chem. Soc., Faraday Trans..

[cit70] Paradas M., Campaña A. G., Jiménez T., Robles R., Oltra J. E., Buñuel E., Justicia J., Cárdenas D. J., Cuerva J. M. (2010). J. Am. Chem. Soc..

[cit71] Hilche T., Younas S. L., Gansäuer A., Streuff J. (2022). ChemCatChem.

[cit72] Zhou C., Zhang B., Hu P., Wang H. (2020). Phys. Chem. Chem. Phys..

[cit73] Snelgrove D. W., Lusztyk J., Banks J. T., Mulder P., Ingold K. U. (2001). J. Am. Chem. Soc..

[cit74] Mills D. A., Xu S., Geren L., Hiser C., Qin L., Sharpe M. A., McCracken J., Durham B., Millett F., Ferguson-Miller S. (2008). Biochemistry.

[cit75] Costentin C., Robert M., Savéant J.-M., Teillout A.-L. (2009). Proc. Natl. Acad. Sci. U. S. A..

[cit76] Costentin C., Louault C., Robert M., Savéant J.-M. (2009). Proc. Natl. Acad. Sci. U. S. A..

[cit77] Costentin C., Robert M., Savéant J.-M. (2007). J. Am. Chem. Soc..

[cit78] Kindermann N., Günes C.-J., Dechert S., Meyer F. (2017). J. Am. Chem. Soc..

[cit79] Carrell T. G., Smith P. F., Dennes J., Dismukes G. C. (2014). Phys. Chem. Chem. Phys..

[cit80] Schreiber E., Brennessel W. W., Matson E. M. (2022). Inorg. Chem..

[cit81] Luo Y.-R. , Comprehensive Handbook of Chemical Bond Energies, CEC Press, Boca Raton, 2007

[cit82] Hillhouse G. L., Bercaw J. E. (1984). J. Am. Chem. Soc..

[cit83] Warburton R. E., Mayer J. M., Hammes-Schiffer S. (2021). J. Phys. Chem. Lett..

[cit84] Castillo-Lora J., Delley M. F., Laga S. M., Mayer J. M. (2020). J. Phys. Chem. Lett..

[cit85] Lyon L. A., Hupp J. T. (1999). J. Phys. Chem. B.

[cit86] Li J.-Q., Meng L., Sprik M., Cheng J. (2020). J. Phys. Chem. C.

[cit87] Lee J., Shizu K., Tanaka H., Nakanotani H., Yasuda T., Kaji H., Adachi C. (2015). J. Mater. Chem. C.

[cit88] CrysAlisPro, version 171.41.97a, Rigaku Corporation, Oxford, UK, 2021

[cit89] Sheldrick G. M. (2015). Acta Crystallogr., Sect. A: Found. Adv..

[cit90] Sheldrick G. (2015). Acta Crystallogr., Sect. C: Struct. Chem..

