# Supplementary material for: Coordination-induced bond weakening of water at the surface of an oxygen-deficient polyoxovanadate cluster
Source: Chem Sci. 2022 Oct 11;13(43):12726–37. doi: 10.1039/d2sc04843d (PMC9645371; doi:10.1039/d2sc04843d)
Supplement: SC-013-D2SC04843D-s001 [file SC-013-D2SC04843D-s001.pdf]

Electronic Supplementary Material (ESI) for Chemical Science.
This journal is © The Royal Society of Chemistry 2022

Electronic Supporting Information (ESI)

Coordination-induced Bond Weakening of Water at the Surface of an Oxygen-deficient Polyoxovanadate Cluster

Shannon E. Cooney, Alex A. Fertig,* Madeleine R. Buisch, William W. Brennessel, and Ellen M. Matson*

Department of Chemistry, University of Rochester, Rochester, New York 14627

Supporting Information Table of Contents:

Figure S1. ^{1}H NMR spectrum of **(calix)V_6O_5 (OH_2)** S2
Figure S2. ^{1}H NMR spectrum of **(calix)V_6O_6** S2
Figure S3. Electronic absorption spectra of **(calix)V_6O_5 (OH_2)** and **(calix)V_6O_6** S3
Table S1. Crystallographic parameters of **(calix)V_6O_5(OH_2)** S4
Table S2. Bond valence sum calculations for **(calix)V_6O_5(OH_2)** S4
Figure S4. ^{1}H NMR spectrum of crude reaction of **(calix)V_6O_6** with hydrazobenzene S5
Figure S5. ^{1}H NMR spectrum **(calix)V_6O_6** + 1 eq H_2NQ over 12 hours S5
Figure S6. ^{1}H NMR spectrum of **(calix)V_6O_5(OH_2)** + 1 eq NQ S6
Figure S7. Cyclic voltammogram of **(calix)V_6O_6** in THF S6
Figure S8. Cyclic voltammogram of the reduction of TEMPO S7
Figure S9. Raw kinetic trace of reaction between **(calix)V_6O_5(OH_2)** and TEMPO S7
Figure S10. Worked up kinetic trace of reaction between **(calix)V_6O_5(OH_2)** and TEMPO S8
Figure S11 ^{1}H NMR spectrum of **(calix)V_6O_5(OD_2)** S9
Figure S12. Electronic absorption spectra of **(calix)V_6O_5(OD_2)** S9
Figure S13. ^{2}H NMR spectrum of TEMPO-D S10
Figure S14. k_{obs} vs. [TEMPO] for the reaction between **(calix)V_6O_5(OD_2)** and TEMPO S10

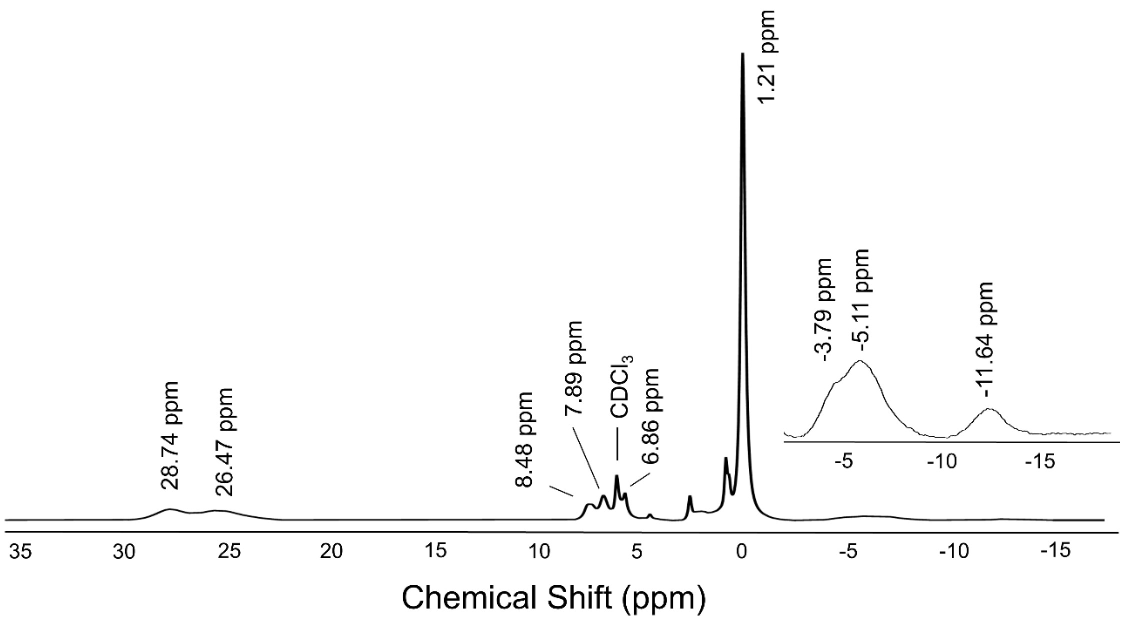

Figure S1. ^{1}H NMR spectrum of **(calix)$V_6O_5(OH_2)$**, collected in $CDCl_3$ at 21 ^oC.

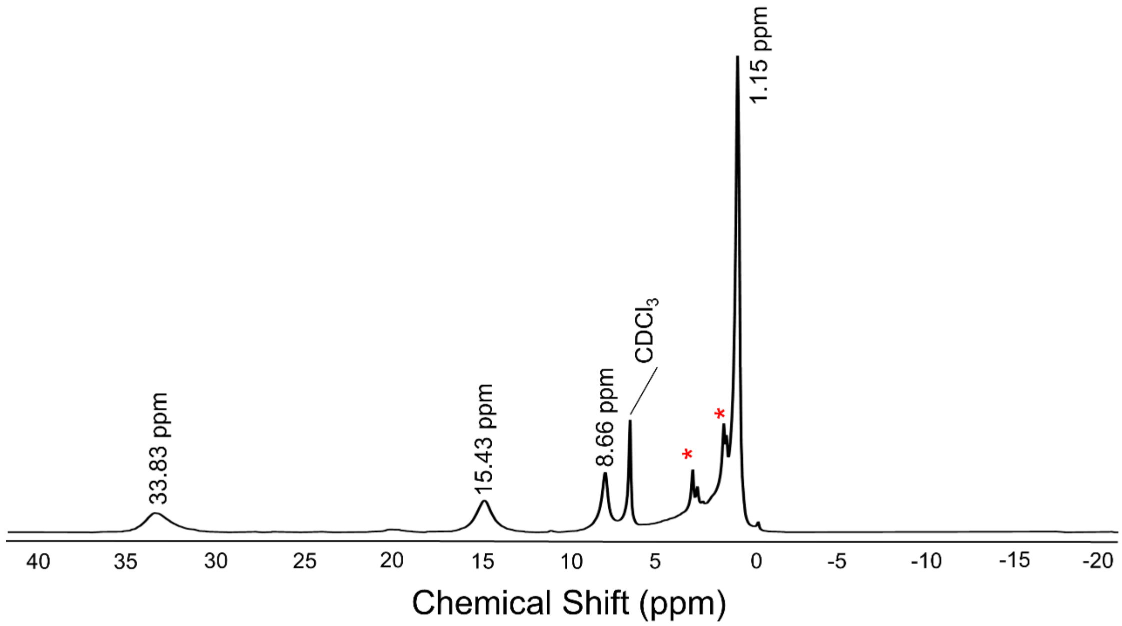

Figure S2. ^{1}H NMR spectrum of **(calix)V_6O_6**, collected in $CDCl_3$ at 21^oC, red asterisk indicates residual THF solvent.

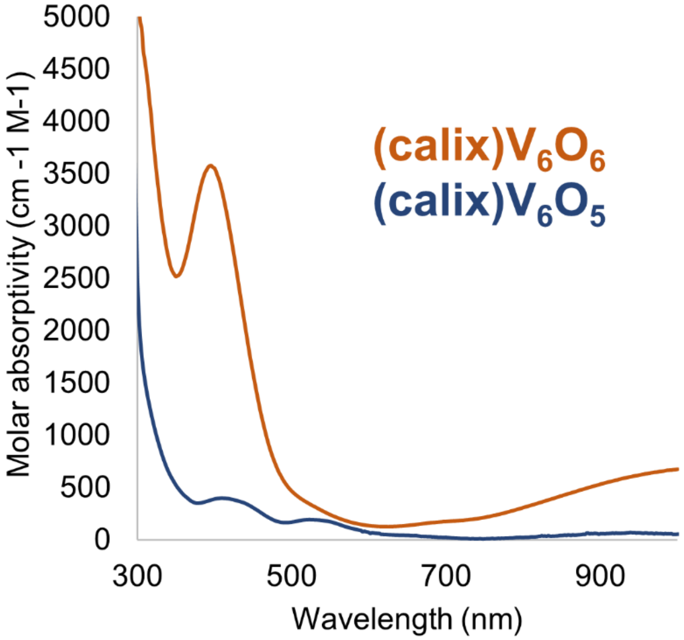

Figure S3. Electronic absorption spectra of **(calix)$V_6O_5(OH_2)$** (blue), and **(calix)V_6O_6** (orange) in tetrahydrofuran.

Table S1: Crystallographic parameters for molecular structure of **(calix)V_6O_5 (OH_2)**.

Empirical formula	$C_{52}H_{76}O_{19}V_6$
Formula weight	1577.57
Temperature / K	173.00(10) K
Wavelength / Å	1.54184 Å
Crystal system	orthorhombic
Space group	*Pbcm*
Unit cell dimensions	*a* = 14.4807(2) Å<br>*b* = 27.8595(5) Å<br>*c* = 18.7836(2) Å<br>α = 90°<br>β = 90°<br>γ = 90°
Volume / $Å^3$	7577.78(19) $Å^3$
Z	4
Reflections collected	48002
Independent reflections	8377 [*R*(int) = 0.0397]
Completeness to theta = 67.684°	99.9%
Goodness-of-fit on F^2	1.038
Final *R* indices [*I*>2sigma(*I*)]	*R*1 = 0.0644, *wR*2 = 0.1861
Largest diff. peak and hole	0.665 and -0.479 e.$Å^{-3}$

Table S2. Bond valence sum (BVS) calculations for crystallographically independent vanadium ions in **(calix)V_6O_5(OH_2)** based on X-ray crystallographic data collected at 100 K. Table reflects the results of BVS calculations using V-O bond valence parameters (r_0) for different oxidation states of vanadium.

**(calix)V_6O_5(OH_2)**	V1	V2	V3	V4	V5
V(III)	**3.194**	3.826	3.921	**3.051**	3.814
V(IV)	3.270	**3.917**	**4.015**	3.124	**3.905**
V(V)	3.513	4.178	4.278	3.359	4.167

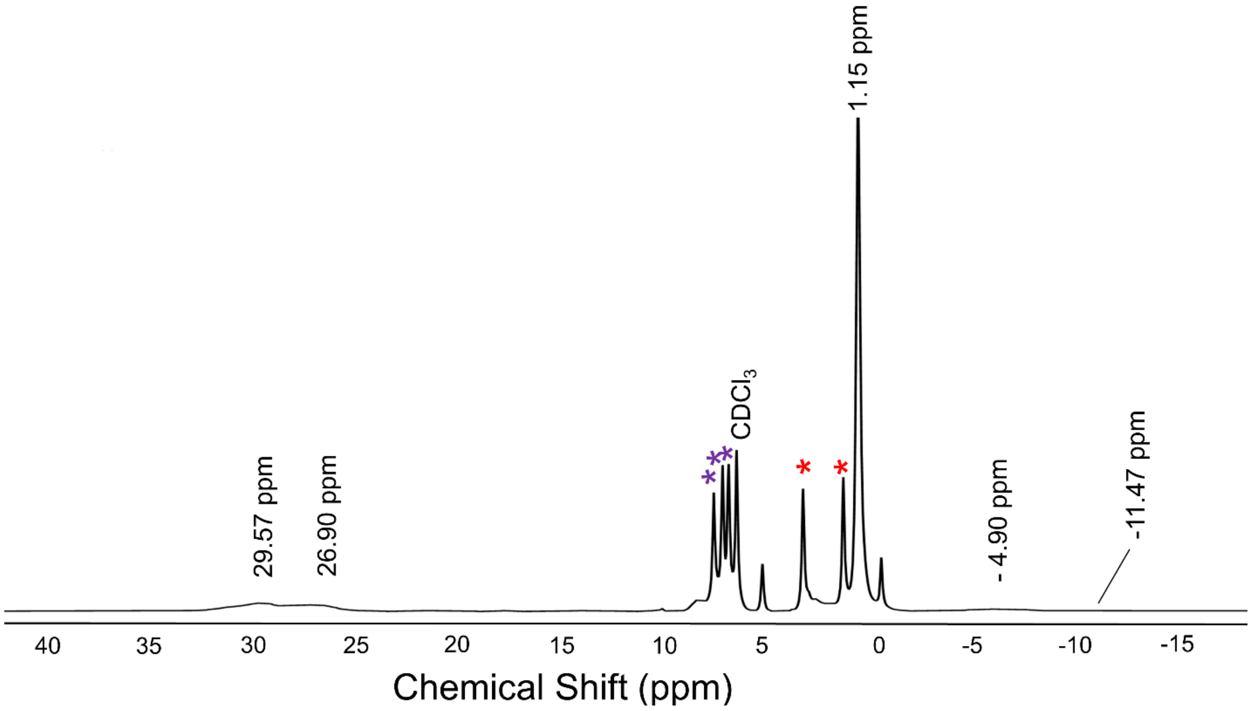

Figure S4. ^{1}H NMR spectrum of crude reaction of **(calix)V_6O_6** with hydrazobenzene after 5 hours at 25 °C. Red asterisks are residual solvent (THF) and purple asterisk indicate azobenzene.

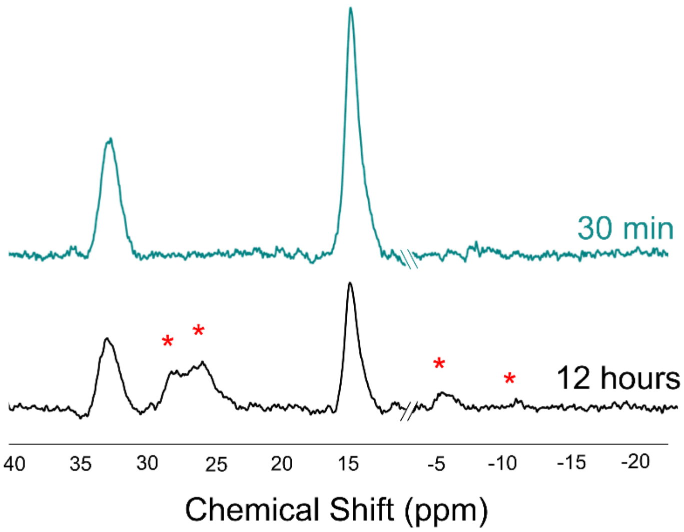

Figure S5. ^{1}H NMR spectrum **(calix)V_6O_6** + 1 eq H_2NQ after 30 minutes (top, teal) in THF-d_8. Reaction reached equilibrium at 25 °C after 12 hours (bottom, black). Red asterisks indicate the formation of **(calix)$V_6O_5(OH_2)$**.

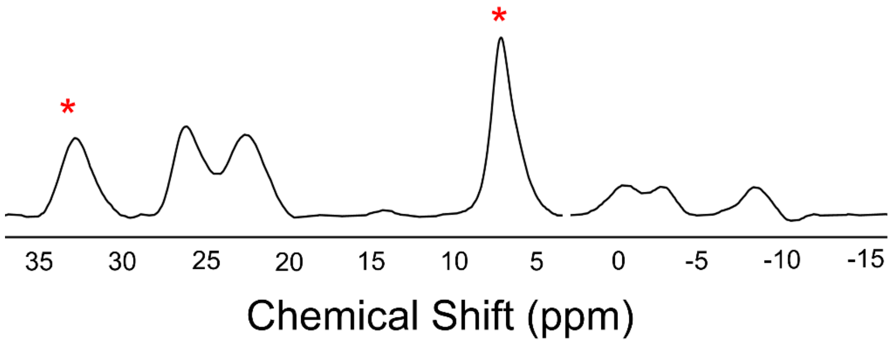

Figure S6. ^{1}H NMR spectrum of **(calix)$V_6O_5(OH_2)$ +** 1 eq NQ in THF-d_8. Red asterisks indicate the formation of **(calix)V_6O_6** after 12 at 25 °C.

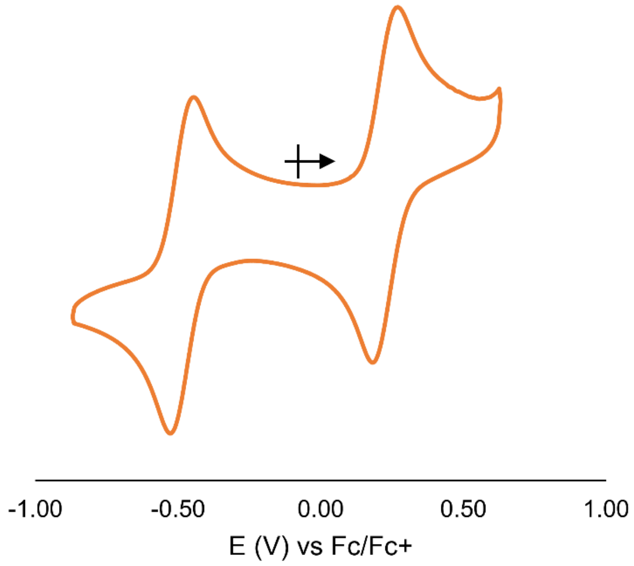

Figure S7. Cyclic voltammogram 1 mM **(calix)V_6O_6** with 100 mM $[NBu_4][PF_6]$ electrolyte at a scan rate of 20 mV s^{-1} in THF.

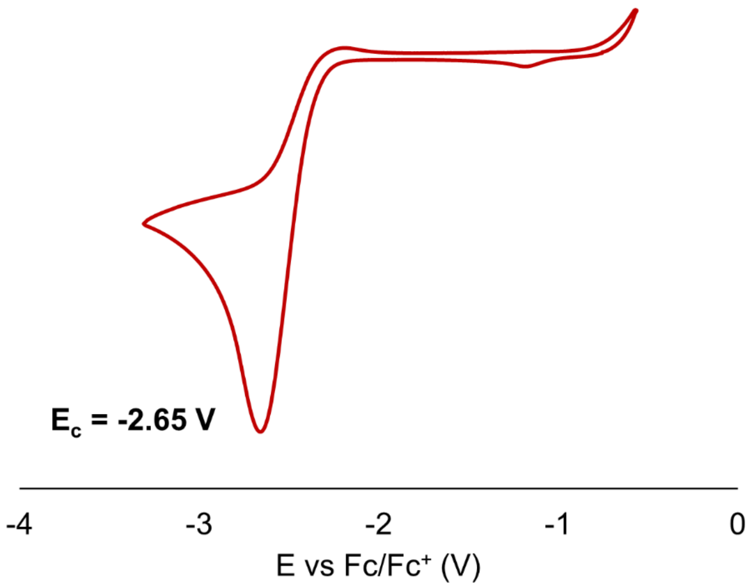

Figure S8. Cyclic Voltammogram of the irreversible reduction of TEMPO in THF vs Fc/Fc^+ with 0.1 M $TBAPF_6$ as a counter electrolyte.

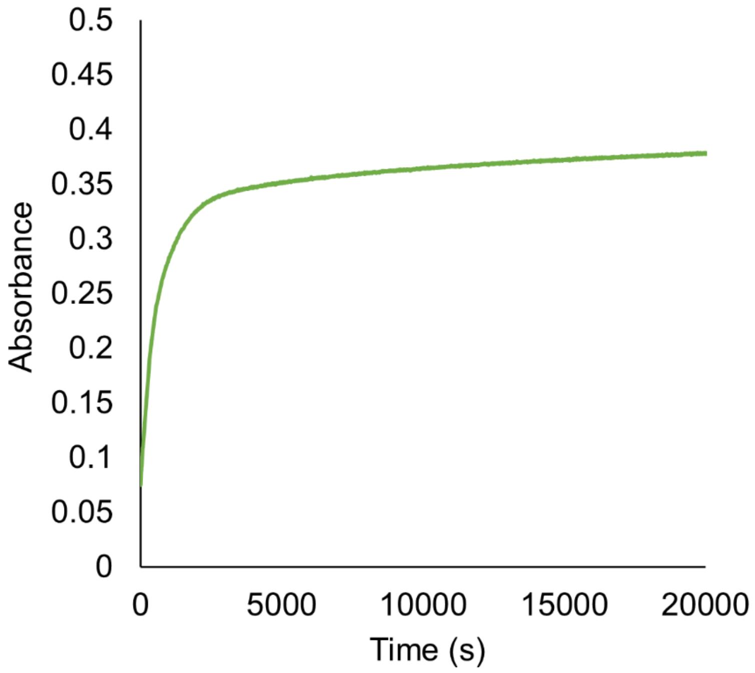

Figure S9. Absorbance trace for the reaction between 0.5 mM **(calix)$V_6O_5(OH_2)$**, and 43 mM TEMPO in THF. The extent of the reaction was monitored at 900 nm. Experiment was performed at 25 ^oC.

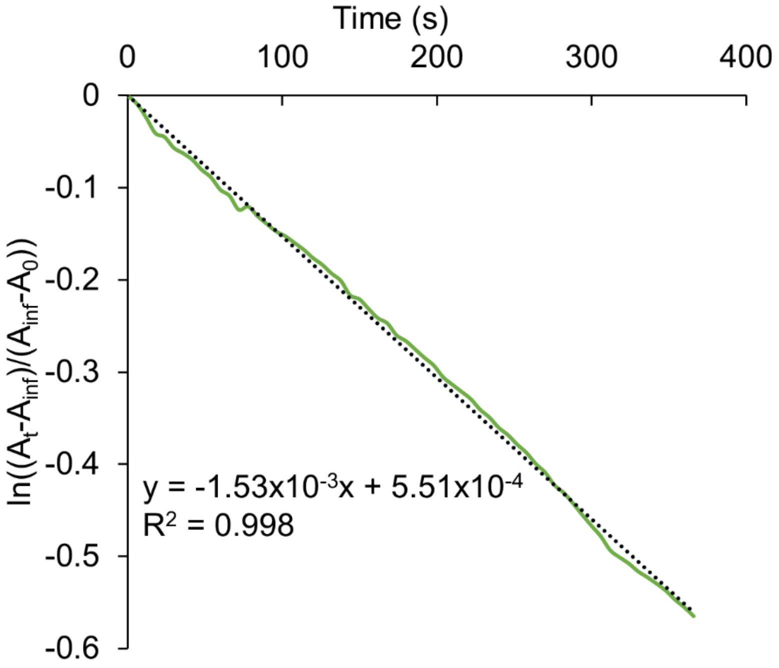

Figure S10. Plot used to obtain the pseudo first-order rate constant for kinetic experiments performed in this work. Plotting $\ln(A_t\text{-}A_{inf}/A_{inf}\text{-}A_0)$ against time results in a linear trend, from which the k_{obs} value can be determined from the absolute value of the slope. A_t = absorbance at time = t, A_{inf} = absorbance at equilibrium, and A_0 = initial absorbance.

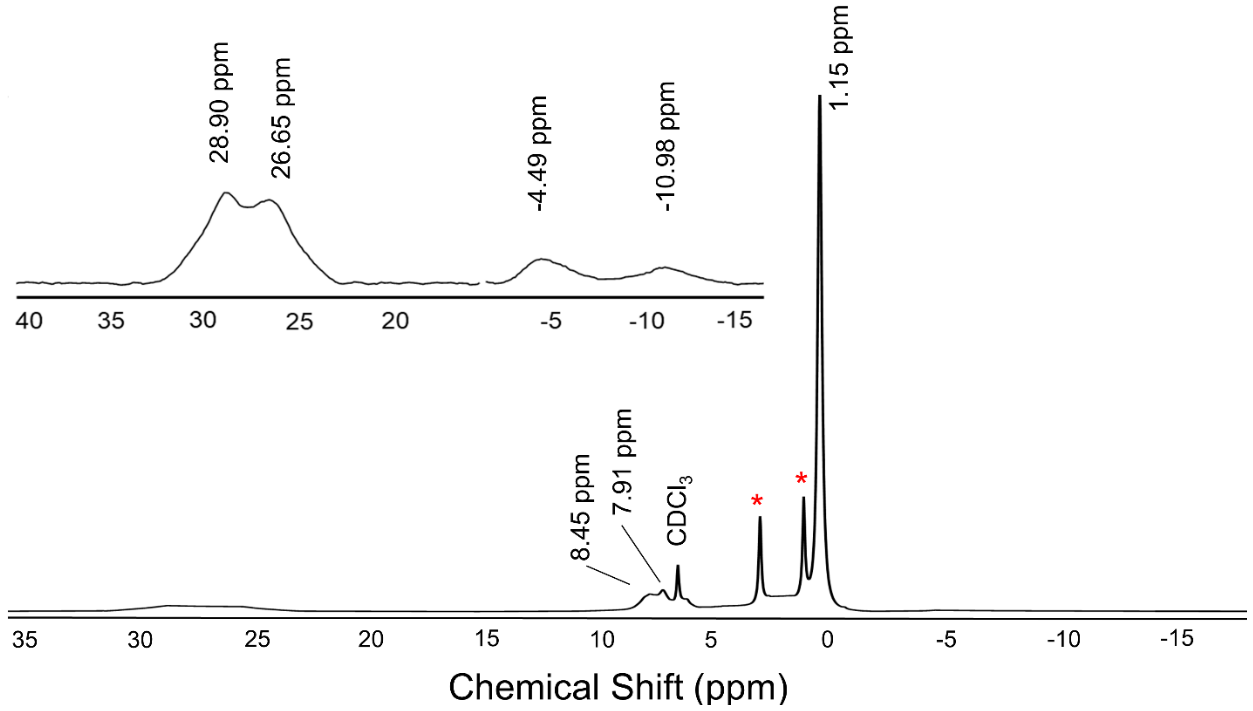

Figure S11. ^{1}H NMR spectrum of **(calix)$V_6O_5(OD_2)$** in $CDCl_3$, red asterisks indicate residual THF.

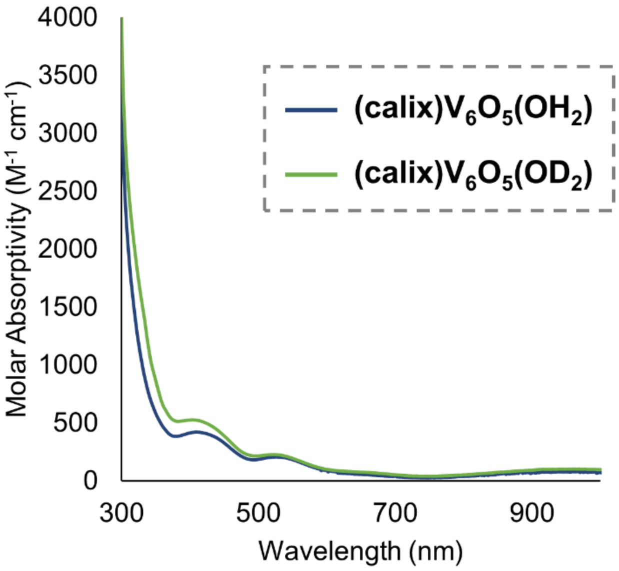

Figure S12. Comparison of the electronic absorption spectra for **(calix)$V_6O_5(OH_2)$** and **(calix)$V_6O_5(OD_2)$**. Both samples were collected in THF at 21 °C. The similarity of both spectra suggests the successful isolation of the deuterated cluster.

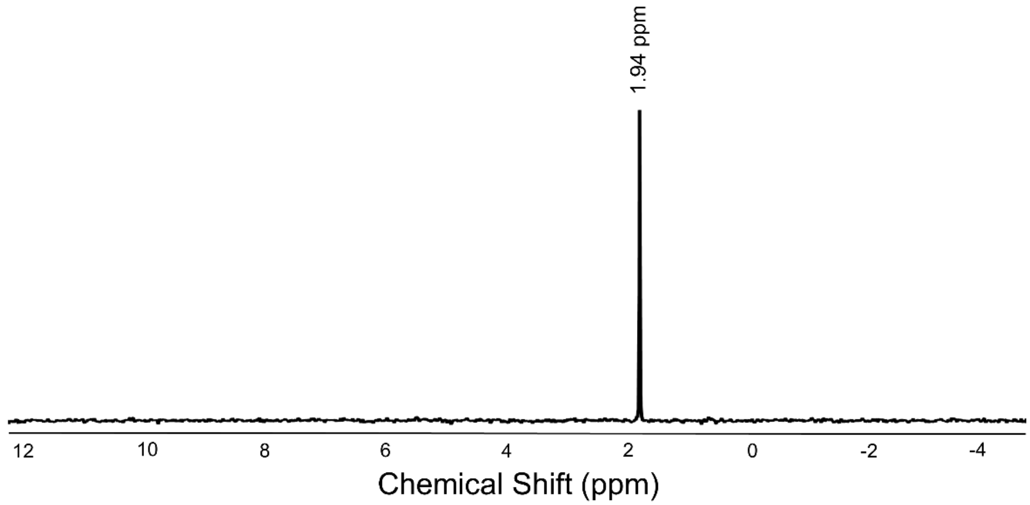

Figure S13. ^{2}H NMR spectrum of TEMPO-D in tetrahydrofuran after a reaction of **(calix)$V_6O_5(OD_2)$** with 2 eq. TEMPO.

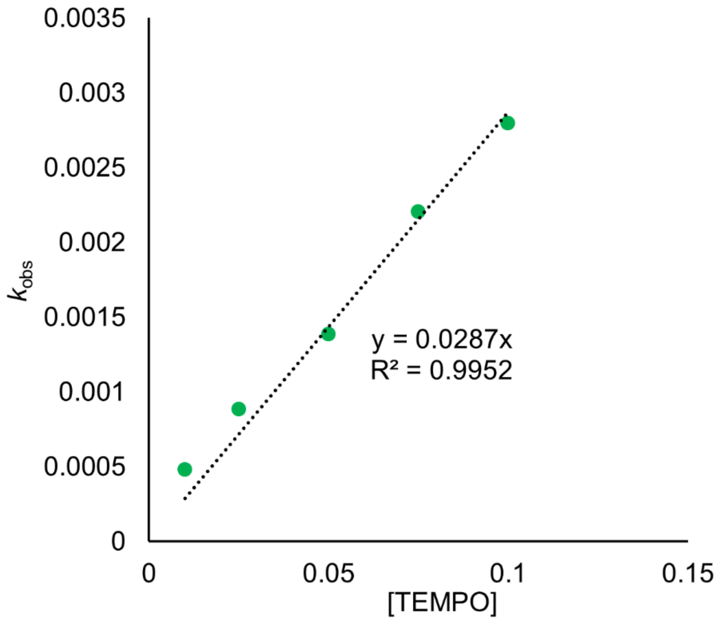

Figure S14. Plot of the observed rate constant, k_{obs}, collected for the reaction between **(calix)$V_6O_5(OD_2)$** and TEMPO against the concentration of TEMPO originally in solution. Each experiment was run with 0.5 mM of **(calix)$V_6O_5(OD_2)$** initially in the solution. The linear trend of the plotted results indicates first order with respect to TEMPO, where the second order rate constant for the reaction, $k_{PCET\text{-}D}$, is determined from the slope of the best fit line with a Y-intercept of 0. Of note, $k_{PCET\text{-}D}$ is equal to ½ the slope of the line in order to account for the two chemically identical D atoms at the reduced cluster.
